# Recent high-resolution Antarctic ice velocity maps reveal increased mass loss in Wilkes Land, East Antarctica

**DOI:** 10.1038/s41598-018-22765-0

**Published:** 2018-03-14

**Authors:** Qiang Shen, Hansheng Wang, C. K. Shum, Liming Jiang, Hou Tse Hsu, Jinglong Dong

**Affiliations:** 1 State Key Laboratory of Geodesy and Earth’s Dynamics, Institute of Geodesy and Geophysics, Chinese Academy of Sciences, Wuhan, 430077 China; 2 Division of Geodetic Science, School of Earth Sciences, The Ohio State University, Columbus, Ohio, 43210 USA; 3 University of Chinese Academy of Sciences, Beijing, 100049 China

## Abstract

We constructed Antarctic ice velocity maps from Landsat 8 images for the years 2014 and 2015 at a high spatial resolution (100 m). These maps were assembled from 10,690 scenes of displacement vectors inferred from more than 10,000 optical images acquired from December 2013 through March 2016. We estimated the mass discharge of the Antarctic ice sheet in 2008, 2014, and 2015 using the Landsat ice velocity maps, interferometric synthetic aperture radar (InSAR)-derived ice velocity maps (~2008) available from prior studies, and ice thickness data. An increased mass discharge (53 ± 14 Gt yr^−1^) was found in the East Indian Ocean sector since 2008 due to unexpected widespread glacial acceleration in Wilkes Land, East Antarctica, while the other five oceanic sectors did not exhibit significant changes. However, present-day increased mass loss was found by previous studies predominantly in west Antarctica and the Antarctic Peninsula. The newly discovered increased mass loss in Wilkes Land suggests that the ocean heat flux may already be influencing ice dynamics in the marine-based sector of the East Antarctic ice sheet (EAIS). The marine-based sector could be adversely impacted by ongoing warming in the Southern Ocean, and this process may be conducive to destabilization.

## Introduction

A considerable challenge associated with rigorous sea level projections in the 21^st^ century is that the dynamics of the Antarctic ice sheet is not sufficiently understood in the context of rapidly warming atmosphere and ocean conditions^1–4^. Studies of Antarctic ice sheet processes since the 1990s using satellite, airborne and *in situ* observations^5–8^ have reported present-day ice sheet changes. The West Antarctic ice sheet has experienced a negative mass balance, extensive dynamic thinning on the periphery^9^, accelerated mass loss^5,8^, and grounding line retreat in the Amundsen Sea sector^10^, all of which have highlighted the long standing concerns about the marine based ice sheet instability^6,11^. In contrast, it is generally considered that the EAIS, which encompasses a larger ice mass, has been in a state of mass equilibrium or has had a slightly positive mass balance over the past two decades^5,12^. However, recent observations have detected thinning in some glaciers on the EAIS, resulting in a negative mass balance in the ice catchments^8,9,13^. Satellite observations and ice-proximal evidence have demonstrated repeated large-scale advances and retreats of glacier termini^14–18^. Ice dynamic studies based on ice sheet modelling also indicate sensitivity to ocean forcing^19–22^. All of these findings suggest that the EAIS has a highly dynamic nature and may be more vulnerable to climatic and oceanography forcing than previously thought. However, the mass balance of the predominantly land-based EAIS remains less clear due to the lack of observable evidence^15,19^. It is also unclear whether the rate of Antarctic ice loss/gain has increased/decreased over the past two decades^23^. Furthermore, the underlying drivers of ice sheet changes remain poorly understood^24^. All these limitations make it difficult to determine the future behaviour of the ice sheet. The key to understanding the dynamics of the Antarctic ice sheet involves more accurately determining its mass budget using extended observations to provide a longer and higher-resolution observational record and improve the understanding of ice sheet evolution, which is crucial for accurate future projections of global sea level^4,6^.

Ice velocity, one of the critical ice dynamic parameters that affects estimates of the ice sheet mass balance and the corresponding sea level rise^25^, has been determined from traditional ground-based measurements (e.g., GPS, electronic distance, etc.) since the 1970s in the Antarctic ice sheet. However, the sporadic and discontinuous observations prohibit the study of the ice sheet mass balance as a whole. Not until recently have glaciologists begun to present a complete picture of ice velocity in Antarctica using multi-satellite interferometric synthetic aperture radar (InSAR) with a long time span (1996–2009)^26^. However, such a snapshot of the ice motion in Antarctica is insufficient for providing clear insight into the spatial and temporal characteristics of ice dynamics. Furthermore, the lack of high-resolution ice velocity data limits a thorough investigation of localized ice dynamics^27,28^, such as crevasse production, the role of ice rises on the stability of ice sheets, etc. These limitations highlight the need for a new set of high-resolution ice velocity observations over Antarctica.

Here, we present two ice velocity maps covering the years 2014 and 2015 for all of Antarctica inferred from Landsat 8 (L8) images acquired by the Operational Land Imager (OLI). The ice velocity data from 2014 and 2015 are then compared with an existing InSAR-derived ice velocity dataset from 2008^29^, and ice discharges are calculated from all three years using a compilation of ice thickness data. Furthermore, the mass balances of the Antarctic ice sheet are estimated by comparing the mass discharges with the latest ice sheet surface mass balance (SMB) data derived from a regional atmospheric climate model (RACMO2.3)^30^ based on an input-output method (IOM)^31^.

## Results

### Ice velocity map

We construct two present-day ice velocity maps spanning the years 2014 and 2015 for all of Antarctica (except for the area south of 82.5° S) inferred from L8 images. These velocity data have the highest spatial resolution of 100 m achieved to date and are assembled from 10,690 scenes of displacement vectors. The vectors are inferred from more than 10,000 orthorectified panchromatic bands with a 15-m spatial resolution acquired by the OLI on Landsat 8 from December 2013 to March 2016 using the optical offset method. Our maps cover nearly all the ice shelves and ice streams and the majority of the ice sheet with an accuracy of 0 to 20 m yr^−1^ (see Methods and Supplementary Discussions 4–8). Our results exhibit a similar pattern in ice velocities compared with published InSAR studies^26,29^, but they yield better coverage at the ice sheet margins. Moreover, the spatial resolution of our velocity maps is 4 to 10 times higher than that of the InSAR-derived ice velocity maps^26,29^.

### Ice dynamics

**Figure 1:**
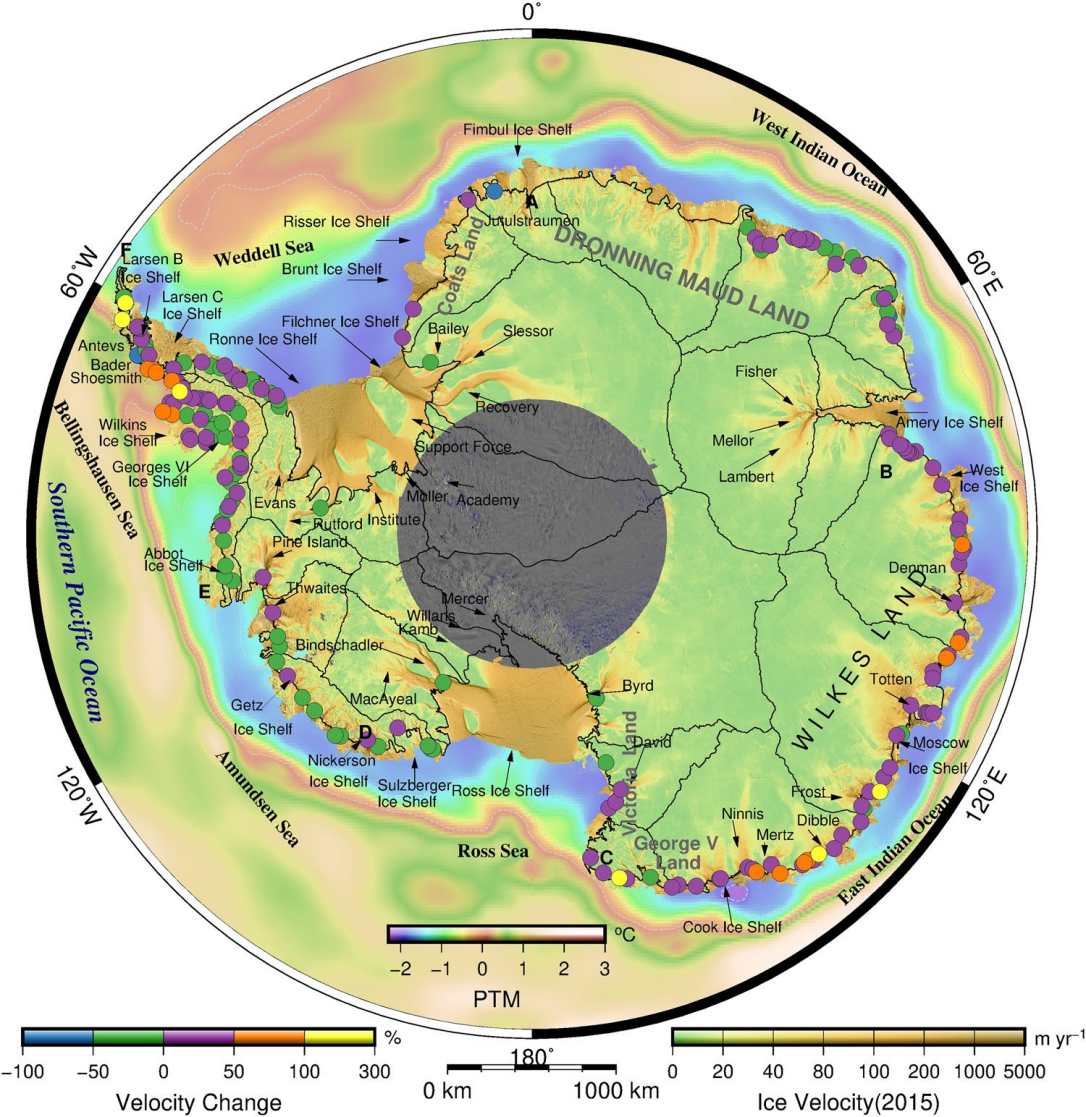
Antarctic ice velocity in 2015 and the velocity change between 2008 and 2015. The mosaic of the Antarctic ice velocity (2015) from L8 panchromatic images from January 2015 to March 2016 is shown here overlaid on a MODIS mosaic of Antarctica (MOA)^34,35^. The magnitude of the ice velocity is coloured on a logarithmic scale and overlaid on gridded potential seawater temperature data (PTM) at a depth of 200 m from the World Ocean Circulation Experiment (WOCE). The velocity changes at grounding lines are calculated for 466 glaciers between 2015 and 2008 and are shown for 211 glaciers with high confidence levels (>2 $\sigma$), which are coloured on a logarithmic scale. The names of selected glaciers and ice shelves are labelled. ‘A’ through ‘F’ delimits the six oceanic sectors. The details of ice velocity changes along grounding lines are presented in Table S1. The solid grey lines delineate major ice divides. This map was created using The Generic Mapping Tools version 5.2.1 (http://gmt.soest.hawaii.edu/)^36^.

We investigate the change in ice velocity of 466 outlet glaciers, accounting for nearly all of the glaciers in Antarctica, using our high-resolution ice velocity map for 2015 and an InSAR-derived map for ~2008^29^ (Table S1 and Supplementary Discussions 4 and 10). Among the studied glaciers, 142 are found to have accelerated, whereas 69 are found to be decelerating at a high confidence level (2 *σ*) (Fig. 1, Supplementary Figs 5, 6 and 9–12). Glacier flow acceleration is still dominant in the Antarctic Peninsula (AP) region. The vast majority of the glaciers in the western AP (WAP) have significantly accelerated (defined as a > 50% change in velocity) compared with the relatively moderate velocity changes for most of the glaciers in the eastern AP (EAP). The rapid changes in WAP were predominately caused by an oceanic driving mechanism^32^ and warmer air temperatures^33^. Conversely, the EAP is surrounded by colder oceanic conditions and lower air temperatures due to the obstacles formed by high mountains. Notably, some glaciers in the northwest AP are excluded from the analysis of glacier dynamics because the InSAR-derived ice velocities of the glaciers are unrealistically low in the grounding line zones. The probable causes are that the InSAR-derived ice velocities have a low resolution and that some data are the results of interpolation due to missing measurements. In west Antarctica, most of the glaciers have slowed since 2008 after accelerating from 1996 to 2006^31^. The fast-flowing Pine Island and Thwaites glaciers, the main outlet glaciers that control the mass drainage from west Antarctica, only exhibit slight accelerations of 1% and 8%, respectively. Although the acceleration of the glaciers is continuing, it is clear that the rate of acceleration is significantly lower than that from 1996–2006 (See Supplementary Figs 7 and 8). Obvious acceleration (approximately 25%) occurred in Victoria Land. In contrast, many of the remaining glaciers that drain into the Ross Ice Shelf, Getz Ice Shelf and Abbot Ice Shelf have decelerated (see Supplementary Figs 5 and 6). The glaciers draining into the Ross Ice Shelf show no significant change or slight deceleration, and this behaviour clearly differs from that of glaciers in Victoria Land. The apparent difference in ice velocity changes between the two regions may be attributed to the warmer Circumpolar Deep Water (CDW), which has intruded the grounding lines in Victoria Land^3^. Additionally, a colder subglacial environment has been maintained by the cold ice shelf meltwater of the Ross Ice Shelf. In East Antarctica, we find that there are strong regional differences, with glaciers with accelerating flows, decelerating flows or no significant change in various regions. In the West Indian Ocean sector, most of the glaciers in Dronning Maud Land and near the Amery Ice Shelf exhibit no significant velocity change, but the glaciers in Enderby Land near Casey Bay and Lutzow-Holm Bay display apparent accelerated velocities. In the East Indian Ocean sector, the vast majority of the glaciers have experienced flow accelerations of up to ~25% (Supplementary Figs 11 and 12). Details of the dynamics of individual glaciers can be found in Supplementary Discussion 10.

### Ice discharges

**Figure 2:**
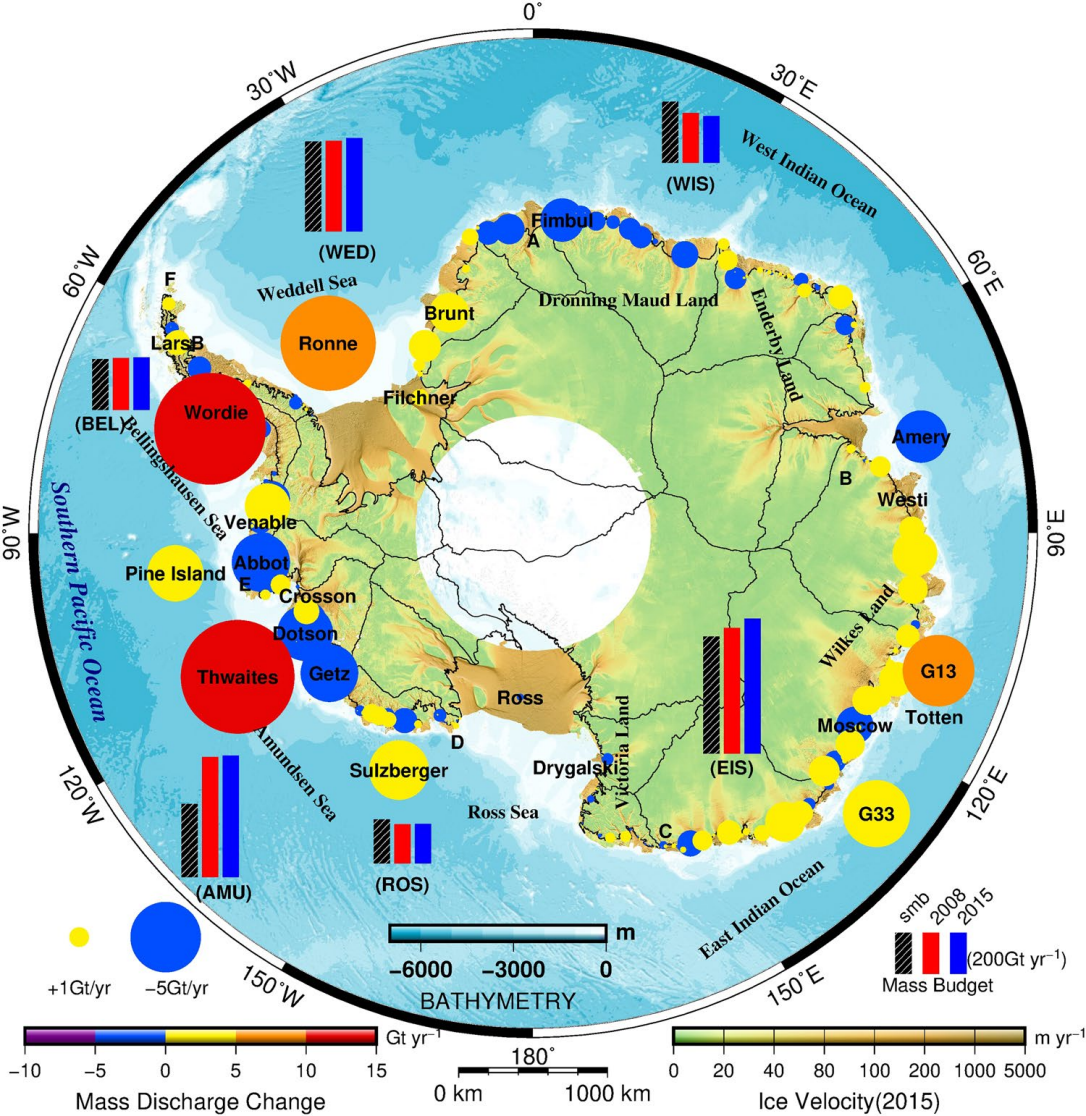
Changes in mass discharges and mass balances across the Antarctic ice sheet between 2008 and 2015. The colour and size of the circles denote the magnitudes of the mass discharge changes for individual glaciers with no ice shelf link and for the combinations of glaciers linked to the same ice shelf. Note that the circles are drawn using variable size scales for clarity. Details about the glaciers can be found in Table S2. In addition, the SMB values and mass discharges in six oceanic sectors in 2008 and 2015 are denoted by black-hatched and coloured bars. The mosaic of ice velocity in 2015 and the ice divides are same as in Fig. 1, and an overlain bathymetric map is shown. The six oceanic sectors include the Ross Sea (ROS), Amundsen Sea (AMU), Bellingshausen Sea (BEL), Weddell Sea (WED), West Indian Ocean (WIS) and East Indian Ocean (EIS). The map was created using The Generic Mapping Tools version 5.2.1 (http://gmt.soest.hawaii.edu/)^36^.

We calculate ice discharges at the drainage basin scale^37^ using ice velocity measurements from 2014 and 2015; the InSAR-derived ice velocity in ~2008 (see Supplementary Discussion 2); a compilation of ice thickness data, mainly derived from our analysis of Cryosat-2 (CS2) radar altimeter measurements associated with Bedmap2^38^; and ice-penetrating radar (IPR) thickness values from multiple campaigns from 2002 to 2014 from the IceBridge project^39–42^ (see Methods and Supplementary Discussion 3). We compare the ice sheet discharges with new SMB data (1979–2014)^30^ to estimate the Antarctic mass balance using the input-output method^31^. The mass discharges across the Antarctic grounding lines^43^ are derived from the flux gate method^44^ using a newly developed procedure (see Methods and Supplementary Discussion 9). Here, we calculate the ice sheet inflow mass for 27 glacier drainage basins^37^ using the new SMB data at a horizontal resolution of 27.5 km produced by the updated regional Atmospheric Climate Model RACMO2.3 (Table S3). Figure 2 shows the mass discharge and mass balance and their changes from 2008 to 2015 covering the entirety of the Antarctic ice sheet. The total mass balance estimates of the Antarctic ice sheet at a constant accumulation rate^45^ during the survey period are −149 ± 71Gt yr^−1^, −241 ± 71 Gt yr^−1^, and −230 ± 71 Gt yr^−1^ in 2008, 2014 and 2015, respectively (Table 1 and S2). These results are comparable to the latest results inferred from GRACE^46^ and Cryosat-2 data^8^ and are consistent with recent InSAR mass balance estimates in 2006^31^. However, our estimated rates are larger than the previous results obtained using ICESat altimetry data^5^. Table S4 shows detailed estimates of the mass balance using altimetry, gravimetry, and IOM in the last several decades. The Amundsen Sea sector had the largest imbalance of −154 ± 27 Gt yr^−1^ in 2015 (similar to the results of a previous study^6^) and accounted for 2/3 of the total imbalance (−230 ± 71 Gt yr^−1^) of the entire Antarctic ice sheet. In addition to the Amundsen Sea sector, another significant negative imbalance (−86 ± 33 Gt yr^−1^) was observed in the East Indian Ocean sector of East Antarctica. In contrast, the West Indian Ocean sector exhibited a clear positive mass balance (51 ± 31 Gt yr^−1^). The Weddell sector exhibited a slight mass gain, whereas the Ross Sea and the Bellingshausen Sea sectors exhibited no significant mass changes. The mass balance values in the Bellingshausen Sea sector are likely underestimated because the summer meltwater is not considered (see Supplementary Discussion 10).

**Table 1:** Mass budgets for the six oceanic sectors of the Antarctic ice sheet. The glacier mass discharge or grounding line flux is denoted by ‘GLF’; the mass balance denoted by ‘Net’ is SMB minus GLF; and the grounding line length is denoted by ‘GLL’. The results for 2014 are given for the period from December 2013 to December 2014, and those for 2015 are from January 2015 to March 2016. The ice sheet area (Area) excludes ice rises and islands to isolate the main ice sheet. The details about the glacier affiliations with the six oceanic sectors can be found in the Supplementary Information.

Oceanic Sector	Area km^2^	SMB Gt yr^−1^	GLF (2008) Gt yr^−1^	GLF (2014) Gt yr^−1^	GLF (2015) Gt yr^−1^	Net (2008) Gt yr^−1^	Net (2014) Gt yr^−1^	Net (2015) Gt yr^−1^	GLL km
Ross Sea (ROS)	2763447	191 ± 12	180 ± 15	181 ± 7	181 ± 8	11 ± 19	10 ± 13	10 ± 14	8334
Amundsen Sea (AMU)	590119	319 ± 24	458 ± 3	474 ± 11	473 ± 13	−139 ± 24	−155 ± 26	−154 ± 27	4481
Bellingshausen Sea (BEL)	206768	221 ± 11	205 ± 2	220 ± 10	218 ± 14	16 ± 11	1 ± 14	3 ± 17	5295
Weddell Sea (WED)	3240372	393 ± 26	434 ± 35	452 ± 33	446 ± 34	−41 ± 43	−59 ± 42	−53 ± 42	12124
West Indian Ocean (WIS)	2544605	267 ± 29	229 ± 11	224 ± 13	216 ± 11	38 ± 31	43 ± 31	51 ± 31	7978
East Indian Ocean (EIS)	2549133	511 ± 32	544 ± 11	589 ± 16	597 ± 9	−33 ± 33	−78 ± 35	−86 ± 33	7213
Total in Antarctica	11894445	1901 ± 58	2050 ± 41	2141 ± 42	2131 ± 42	−149 ± 71	−240 ± 71	−230 ± 71	45425

### Mass balance

**Figure 3:**
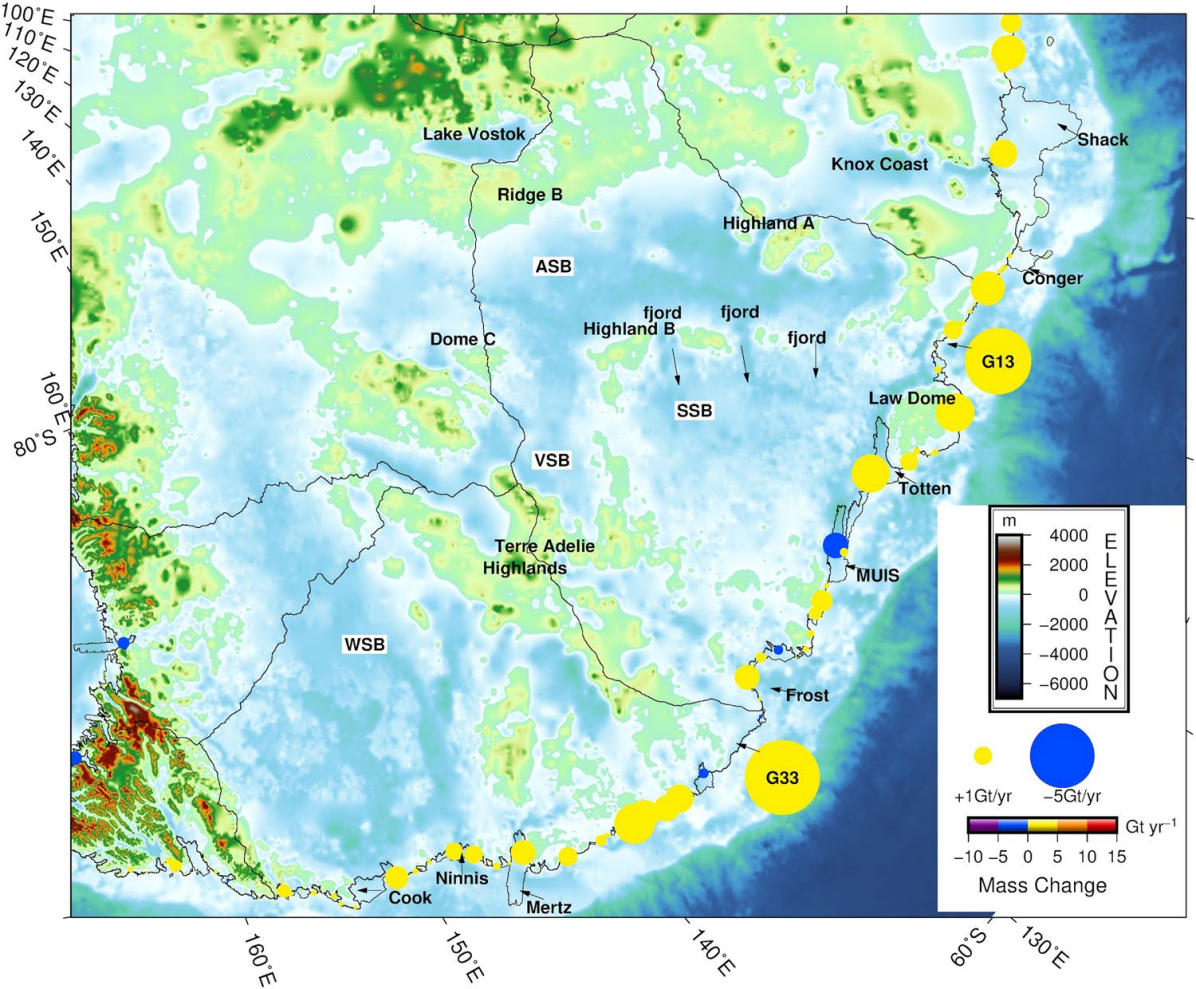
Bed topography of Wilkes Land, East Antarctica^38^. Coloured circles show the mass balance changes between 2008 and 2015 for individual glaciers with no ice shelf link and for glacier combinations linked to the same ice shelf. ASB: Aurora Subglacial Basin, VSB: Vincennes Subglacial Basin, VST: Vanderford Subglacial Trench, SSB: Sabrina Subglacial Basin, and WSB: Wilkes Subglacial Basin. The subglacial basin data are from Young *et al*. (2012). The map was created using The Generic Mapping Tools version 5.2.1 (http://gmt.soest.hawaii.edu/)^36^.

We further analysed the change in the mass balance of the Antarctic ice sheet from 2008 to 2015 (Fig. 2). The mass balance decreased by 54% between 2008 and 2015, reaching a rate of −230 ± 71 Gt yr^−1^ in 2015 compared with −149 ± 71 in 2008, which shows that mass loss from the Antarctic ice sheet is still accelerating. This finding is similar to the results of many previous studies^46–49^. A pronounced change in the mass balance occurred only in the East Indian Ocean sector, where the change reached −53 ± 47 Gt yr^−1^. In addition, we found that the mass discharge from the East Indian Ocean sector increased by as much as 53 ± 14 Gt yr^−1^, which is attributed to unexpected widespread glacier acceleration in Wilkes Land, East Antarctica. The underlying cause for this increased mass discharge is likely linked to the incursion of warm CDW towards the outlet glacier termini^50^, as well as sea ice reduction and break-up^18,51^. Recent oceanographic observations have confirmed that warm water has intruded into the cavities beneath the Totten Ice Shelf through deep channels, as in West Antarctica and the AP^50^. In Wilkes Land, the large mass discharge increases, together with anomalous glacier retreat^18^, contemporary thinning along the margins^3^, grounding line retreat^52^ and unstable inland-sloping bedrock topography, suggest potential instability in the marine-based sector of the EAIS under warmer ocean currents^6,53^. Once the marine sector began an unstable retreat, as has occurred for the West Antarctic ice sheet, the likelihood of rapid mass loss increased under unstable bedrock configurations, partially due to the sensitivity to oceanic conditions^19,20,50,54,55^. This situation would increase the possibility of abrupt and irreversible ice loss in the marine sector. However, due primarily to the current lack of adequate observational evidence and insufficient understanding of these processes, process-based modelling of the instability projections of marine sector glaciers remains elusive. Consequently, the lack of knowledge regarding glacier instability in this region considerably increases the difficulty of future global sea level projection^2^. Of particular importance are the parts of the EAIS that are underlain by extensive marine-based subglacial basins. The Aurora Subglacial Basin (ASB) in western Wilkes Land is located to the northeast of elevated Dome A and Ridge B on the Antarctic ice sheet (Fig. 3), and it drains ice towards the Sabrina Coast via the Totten glacier. The ASB is overlain by 2–4.5 km of ice and holds an ice mass equivalent to a 9-m sea level rise. IPR data were used to identify a series of deep topographic troughs (more than 1 km below sea level) within a mountain block landscape oriented nearly orthogonal to the modern margins^54^. The increased mass discharge at the margins of the ice sheet may trigger the instability of the ASB with a deep landward-dipping subglacial topography. This process has occurred many times throughout the palaeo-climatic history of Antarctica and has significantly contributed to sea level change^54^. The Wilkes Subglacial Basin (WSB) of eastern Wilkes Land, which holds an ice mass equivalent to a 19-m sea level rise^19^ and drains through marginal glaciers (e.g., Cook and Ninnis ice streams) near George V Land, functions as an ice plug to support the marine-based WSB. However, we find that these glaciers exhibit evidently increased mass discharges, which may already be influencing ice dynamics in the marine-based sector of the EAIS. In contrast, the other five sectors exhibit no significant changes in mass discharge. Notably, the increased mass discharges over the last seven years in the Pine Island (basin 21) and Thwaites catchment (basin 22), West Antarctica, and the AP are found to be 13 ± 4 Gt yr^−1^, 15 ± 54 Gt yr^−1^, and 21 ± 10 Gt yr^−1^, respectively. The values are notably less than the previous estimates of 46 ± 5 Gt yr^−1^, 46 ± 23 Gt yr^−1^ and 29 ± 13 Gt yr^−1^, respectively, from 1996–2006^31^. However, the exact underlying causes are unclear.

In this contribution, we constructed two Antarctic-wide high spatial resolution (100 m) ice velocity maps covering the years 2014 and 2015. These maps provide a new tool for obtaining a more comprehensive understanding of the current ice dynamics in the area. We found a significantly increased mass discharge of 53 ± 14 Gt yr^-1^ in the East Indian Ocean sector over the last seven years. This increase was attributable to widespread acceleration in the glaciers of Wilkes Land, East Antarctica. The other five oceanic sectors did not exhibit distinct changes in mass discharge; however, the contemporary view is that present-day increased mass loss primarily originates from West Antarctica and the AP. Over the time period of our study, the Antarctic ice sheet as a whole exhibited an increased mass loss of 81 ± 100 Gt yr^−1^, which was equivalent to an increased mass loss of 54% since 2008 (−149 ± 71 Gt yr^−1^). More importantly, we found that Wilkes Land in the East Indian Ocean sector contributed most to increased mass loss, reaching 53 ± 47 Gt yr^−1^. The significant increase in mass discharge in Wilkes Land suggests the potential risk of the destabilization of the marine-based sector of the EAIS, which has large subglacial basins, inland-sloping bedrock and deep troughs, i.e., an instable bedrock configuration similar to that in West Antarctica. Our new high-resolution ice velocity maps together with historic InSAR ice velocity data allow us to determine the first continent-wide changes in ice velocity and ice discharge over the past seven years. These results contribute to our understanding of Antarctic-wide ice dynamics and can potentially improve ice sheet modelling and future sea level projections.

## Methods

### Optical offsets

The optical image correlation technique is used to determine the horizontal ice displacement field. This technique measures the displacement pixel-by-pixel between optical satellite images acquired at different times. The local frequency content is calculated at the same location in both images within a specific sliding window. Horizontal displacement maps, including North-South (N-S) and East-West (E-W) components, are then determined from the phase shift of the low-frequency content using the convention that eastward and northward are positive. The technique allows us to co-register optical satellite images with unprecedented accuracy to produce high-quality displacement measurements between pairs of images. Specifically, the approach enables us to resolve subpixel displacements of less than 1/20 of the Landsat 8 pixel resolution (15 m) at a high signal-to-noise ratio (SNR), which is generally greater than 0.9. All processes are performed using the COSI-Corr (Co-registration of Optically Sensed Images and Correlation) software package developed at the California Institute of Technology^56^, which is freely available from http://www.tectonics.caltech.edu/slip_history/spot_coseis/index.html. Further information about the methodology can be found in the Supplementary Information.

### Ice velocity

The ice velocity is obtained by applying the optical offset method to orthorectified panchromatic bands with a 15-m spatial resolution acquired by the OLI on the Landsat 8 satellite from the U.S. Geological Survey (USGS) Earth Resources Observation and Science (EROS) Center between December 2013 and March 2016. To enhance the accuracy of the mosaicked ice velocity, all displacement scenes are stacked based on a set of algorithms developed for the seamless generation of ice velocity mosaics under the consideration of absolute calibration. Simultaneously, the procedure can also output spatially varying error estimates for the ice velocity with magnitudes of 0–20 m yr^−1^. For InSAR-derived ice velocity, we used annual ice velocity data from 2007/08 and 2008/09 to produce a full mosaic of the entire Antarctic ice sheet. Further information about the methodology can be found in the Supplementary Information.

### Mass budget (input-output)

The mass budget method relies on quantifying the difference between ice sheet mass gained (input) through snowfall, with considering sublimation and melt water runoff, and the perimeter ice discharge flux (output). The ice sheet mass gained, which is also called the net SMB, is generally simulated using a regional atmospheric climate model^30^. The SMB values are estimated for the 27 glacier drainage basins. The ice discharge flux across grounding lines is calculated using the rigorous flux gate method^44^ in combination with ice velocity values, a compilation of CS2 ice thicknesses, Bedmap-2 ice thicknesses, and IPR track measurements from the IceBridge project. CS2 ice thickness data are adjusted for ice flux estimates in corresponding periods using 18-year observations of ice shelf thickness changes at a 27-km spatial resolution^57^.

### Data availability

The data used in this paper include ice velocity data, ice thickness data, optical satellite images and grounding line products. The ice velocity data are from http://nsidc.org/data/docs/measures/nsidc0484_rignot/, http://nsidc.org/data/nsidc-0720, and http://nsidc.org/data/velmap/. To obtain the velocity products, please contact the author. The ice thickness products are provided by the Bedmap programme at http://www.antarctica.ac.uk/bas_research/data/ access/bedmap/download/ and by IceBridge (IPR data) at http://nsidc.org/data/docs/daac/icebridge/irmcr3/, https://nsidc.org/data/docs/daac/icebridge/brmcr2/, http://nsidc.org/data/ir1hi2, and https://nsidc.org/data/ir2hi2. The optical satellite images are from http://glovis.usgs.gov and http://earthexplorer.usgs.gov/ for Landsat. Other data are from http://icesat4.gsfc.nasa.gov/cryo_data/ant_grn_drainage_systems.php for Antarctic drainage basins, http://nsidc.org/data/atlas/news/antarctic_coastlines.html for the MOA grounding line and coastline, http://nsidc.org/data/nsidc-0489 for the ASAID project grounding line, http://nsidc.org/data/docs/measures/nsidc0498_rignot/ for the DInSAR grounding line, http://pangaea.de/ for the grounding line provided by Depoorter *et al*.^43^, http://nsidc.org/data/nsidc-0082 for the Antarctica DEM, http://www.staff.science.uu.nl/ for the FDM and SMB data, http://nsidc.org/data/docs/agdc/nsidc0280/index.html for MODIS Mosaic of Antarctica^58^, and http://woceatlas.tamu.edu/ for the potential temperature of seawater and bathymetry.

## Electronic supplementary material

Supplementary information

Supplementary Tables
