# Supplementary material for: Recent high-resolution Antarctic ice velocity maps reveal increased mass loss in Wilkes Land, East Antarctica
Source: Sci Rep. 2018 Mar 14;8:4477. doi: 10.1038/s41598-018-22765-0 (PMC5852037; doi:10.1038/s41598-018-22765-0)
Supplement: Supplementary file 1 — Supplementary information [file 41598_2018_22765_MOESM1_ESM.pdf]

Supplementary Information:

Recent high-resolution Antarctic ice velocity maps reveal increased mass loss in Wilkes Land, East Antarctica

Qiang Shen[1,3], Hansheng Wang[1,3], C. K. Shum[2,1], Liming Jiang[1,3], Hou Tse Hsu[1,3], Jinglong Dong[1,3]

[1]State Key Laboratory of Geodesy and Earth's Dynamics, Institute of Geodesy and Geophysics, Chinese Academy of Sciences, Wuhan 430077, China

[2]Division of Geodetic Science, School of Earth Sciences, Ohio State University, Columbus, Ohio 43210, USA

[3]University of Chinese Academy of Sciences, Beijing 100049, China

Correspondence to: Qiang Shen (cl980606@ whigg.ac.cn)

Supplementary Tables

Tables S1 to S4 (in separate Excel file)

Table S1. Ice velocities and the associated changes in glaciers and ice shelves. The velocity of a glacier is sampled at the intersection of the grounding line and the profile of the ice flow centre line. The ice-shelf velocity is observed at the intersection of the coastline and the profile.

Table S2. Glacier ice discharges and the associated changes.

Table S3. Basin surface mass balance (SMB) estimates.

Table S4. Mass balance estimates from previous studies. The majority of previous results are from Shepherd et al. (2012).

Supplementary Discussion

1 Supporting data

Based on the approach of Depoorter[59], we define six oceanic sectors: the Ross sea (ROS), Amundsen Sea (AMU), Bellingshausen Sea (BEL), Weddell Sea (WED), West Indian Ocean (WIS and the East Indian Ocean (EIS). Additionally, the method of Zwally et al. (2012) is used to define the glacier basins. Any glacier basin that crosses two oceanic sectors is assigned to the oceanic sector where it is mostly distributed. For example, the Sulzberger catchment and the Batch catchment are assigned to the AMU sector and the BEL sector, respectively.

The grounding lines of the Antarctic ice sheet are obtained from a synthetic compilation of published grounding lines[59]. Since some grounding lines have retreated, especially in the Frost glacier, partial glaciers in the Western Antarctic Peninsula (WAP)

and fast flowing glaciers, the published grounding lines were shifted inland to ensure that ice thickness and ice velocity measurements were available for ice flux estimates.

The ice fluxes (ice mass discharges) were calculated for individual glaciers or ice streams. To investigate the possible buttressing of an ice shelf to the relevant glaciers, all these glaciers were treated as a whole in the mass discharge estimates. For the complete survey of the ice fluxes, the ice fluxes across the grounding lines not fringed by ice shelves were also calculated in this study, whereas previous studies used only extrapolated data for non-surveyed areas[59,60] (Table S2). Additionally, all results are shown with 1σ uncertainty.

2 Data sources

Here, we collected Landsat 8 (L8) orthorectified panchromatic bands with a 15-m spatial resolution from December 2013 to March 2016 to infer the present-day ice velocities of the Antarctic ice sheet. The images were acquired by the Operational Land Imager (OLI) on L8 and are managed by the United States Geological Survey (USGS) Earth Resources Observation and Science (EROS) Data Center. L8 is the eighth satellite in the Landsat missions and was launched on February 11, 2013. The satellite provides a continuous series of land and ice surface observations with a 16-day revisit cycle. The OLI has improved radiometric performance in 12-bit quantization and can distinguish subtle contrast variations over bright targets[61,62], such as those of the Antarctic ice sheet covered only by snow or ice with high reflectivity. Rigorous calibration and orbital control contribute to the resulting high-quality visible and infrared images. The OLI is calibrated to <5% uncertainty in absolute spectral radiance and an ~8-m geodetic

accuracy (circular error at 90% confidence (CE90))[63]. High radiometric resolution, high geodetic accuracy and the large quantity of available observation data have made it possible to determine continent-wide ice velocities for the Antarctic ice sheet[61,64].

The Level 1 terrain-corrected (L1GT) products packaged in geographic tagged image file format (GeoTIFF) in 16-bit greyscale are used to produce the ice velocities of Antarctica. The L1GT data in Antarctica are terrain orthorectified using the Radarsat Antarctic Mapping Project version 2 Digital Elevation Model (RAMP V2 DEM). The geometrically corrected products have minimal distortions relative to the sensor (e.g., view angle effects), satellite (e.g., attitude deviations from nominal), and Earth (e.g., rotation, curvature, and relief). Radiometric corrections are applied to remove relative detector differences, dark current bias, and some other artefacts. A complete L1GT product consists of 13 files: the 11 band images, a product-specific metadata file, and a Quality Assessment (QA) band. In our study, only the panchromatic band, specific metadata file and QA band are used. The specific metadata are used to obtain the cloud ratio as a criterion (40%) to select images for ice velocity extraction. The QA band is used to identify the spatial distributions of cloud and water, which are masked in displacement scenes. In total, more than 10,000 scenes are selected to produce ice velocities across Antarctica based on visual interpretation and the cloud cover ratio. The projection of ice velocity data is polar stereographic with a true latitude of –71°. The reference ellipsoid used is the WGS84 model. In addition, to compare ice flux and mass balance values among different periods, we do not use the mosaic of ice velocity because the synthetic aperture radar (SAR) data are used over a long time span[65]. In

contrast, annual interferometric synthetic aperture radar (InSAR)-derived ice velocity data (1-km spatial resolution) inferred from multi-satellite InSAR data sets are used[64]. The 2007/08 and 2008/09 ice velocity data are combined to produce the full mosaic of Antarctic ice velocity. The mosaic covers more than 98% of the Antarctic grounding line zone, and the missing data in remaining areas are filled by 2006/07 ice velocity data. The majority of InSAR data used here are from 2007 to 2009; the InSAR-derived ice velocity is therefore assumed to be circa 2008 during the mass budget assessment[64].

To assess the accuracy of our ice velocity results, we also collected *in situ* measurements[66-72] compiled and managed by the National Snow & Ice Data Center (NSIDC). The *in situ* measurements of ice velocity were obtained from a variety of methods, such as differential GPS information, electronic distance measurements and triangulation chain surveys. The *in situ* data in the Lambert-Amery basin, the Siple Coast, and the Mizuho Plateau of Queen Maud Land were mainly obtained from 1988 to 2008, 1984 to 1998, and 1969 to 1978, respectively. Note that we compared *in situ* measurements only in the slow-flow regions where ice velocities are less than 100 m yr^{-1} and thus assumed to have no significant secular changes.

3 Ice thickness

We followed the method of Chuter et al. (2015)[73] and recalculated the ice shelf thickness using the Cryosat2 (CS2) altimetry measurements acquired from 2010 to 2017. The glacier mass discharges (GLFs) in ice shelves are estimated using the CS2 ice thickness rather than Bedmap2 because the Bedmap2 ice thickness in ice shelves is inferred from the European Remote sensing satellite (ERS-1), ICESat data and the

interpolated grounded ice thickness for continuity at the grounding line. In areas other than the ice shelves, the Bedmap2 and ice-penetrating radar (IPR) thickness from multiple campaigns between 2002 and 2014, in combination with ice velocity vectors, are used to calculate the ice fluxes. The IPR data are from multiple radar sounder instruments from multiple campaigns of NASA's Operation IceBridge (OIB) project, including the Hi-Capability Radar Sounder (HiCARS) instrument[74,75] and the Multichannel Coherent Radar Depth Sounder (MCorDS)[76,77].

4 Feature tracking method

To determine the horizontal displacement vectors due to ice motion, we use a feature tracking method[78-80], also known as the phase shift method. The orthorectified L8 images are directly used to produce the displacement vectors based on the co-registration (or cross-correlation) method. The method produces the displacement vectors with a low-frequency phase shift technique as calculated by a Fourier-based frequency correlator[80]. These calculations are repetitively produced within a specific sliding window (or patch) for paired images. The result is given by a three-band file consisting of an E-W displacement map (positive towards the East), a N-S displacement map (positive towards the North), and the signal-to-noise ratio (SNR) band as an indicator of the quality of the measurement. The technique can resolve sub-pixel displacements of less than 1/20 of the pixel resolution at a high SNR, which is generally greater than 0.9. The feature tracking method is automatically implemented based on the COSI-Corr (Co-registration of Optically Sensed Images and Correlation) software package developed at the California Institute of Technology[80].

Specifically, the feature tracking process includes two stages. The first stage (namely, coarse co-registration) involves approximate estimates of the pixelwise displacement between two patches. In general, if noisy images or large displacements are expected, a larger initial sliding window should be used. In this study, the size of initial sliding window varies from 64 to 256 pixels in both the X- and Y-directions according to a priori knowledge from the InSAR-derived Antarctic ice velocity and the time interval between two paired images. Once the initial displacements are estimated, the second stage is fine co-registration to retrieve the subpixel displacement using a smaller window. The new size of 32×32 pixels is tentatively adopted to yield reliable estimates of the displacement at densely independent points. Other parameters of the frequency correlator include the step sizes between sliding windows in both the X- and Y-directions (in pixels), the frequency masking threshold, the number of iterations need to meet robustness requirements, resampling and gridded output. The step size is set to a constant value of 7 pixels in each dimension or approximately a 100-meter spatial resolution. The frequency masking threshold of 0.9 is adopted as the optimum value, as recommended in a previous study[80].

5 Quality control for displacement vectors

Generally, the frequency-based co-registration method is more accurate compared with statistical methods, but it is more sensitive to noise contamination. L8 images can minimize the results of good radiometric and geodetic performances, but decorrelation still exists due to large ground motion, the lack of measurable ground features (such as crevasses or rises), sensor noise, and topographic artefacts (which can lead to imprecise

orthorectified data).To overcome these problems, we devise three steps to enhance the signal and exclude unreliable measurements. First, we suppress the noise in each displacement scene using an adaptive filter and a median filter. The adaptive filter is the local sigma filter[81], which features a filter size of 9 pixels and a sigma factor value of 2. A median filter is further applied to remove "salt and pepper" noise in ice displacement scenes. Second, the areas covered by clouds and water are excluded from the displacement scenes using the QA band[63]. In the QA band, each pixel contains a 16-bit integer that represents bit-packed combinations of surface, atmosphere, and sensor conditions at different confidence levels. The pixels covered by clouds and water in paired images are unpacked from the QA band using the procedures we have developed, and the pixels marked as clouds and water at high confidence levels (67–100%) are used to build a mask layer. These pixels are then masked in displacement scenes. Note that the identification of cirrus clouds is problematic in raw images based on our analysis because the radiometric characteristics of ice and cirrus clouds are generally indistinguishable. Here, we only use the clouds to build a mask layer. Third, since frequency correlation easily generates errors at the edges of displacement scenes, the results of the displacement vectors are neglected in edge regions.

6 Ice velocity extraction

Cloud contamination is a major challenge in ice velocity estimation using optical images. To overcome this problem, we process all image pairs using a time interval of one year as a temporal baseline with a minimum repeat cycle of 16 days in the Worldwide Reference System (WRS-2). Some images in adjacent paths in WRS-2 are

also paired to produce ice velocities in some void areas where there are no valid scenes with the same path and row available. The one-year time interval adopted is derived from our experiments. When the time interval is greater than one year, decorrelation may appear due to large surface motion or geomorphic changes. Finally, 10,690 image pairs are selected from more than 10,000 scenes of L8 panchromatic images and are processed to generate ice velocity estimates.

Despite the geometric accuracy improvement provided by L8, the residual geolocation errors (~8 m in CE90) of the L8 panchromatic band contribute most to uncertainties in ice velocity products. These errors will lead to an offset between displacement scenes and should be removed[61]. In fact, offset tuning is often called the absolute calibration of ice velocity data. In Antarctica, absolute calibration is a challenging issue because the ice is active almost everywhere and available rock outcrops are extremely scarce. Here, we use the InSAR-derived Antarctic velocity map to determine the relatively stagnant areas (i.e., areas with ice velocities of <5 m yr^{-1}) for the absolute calibration of our ice velocity estimates.

There are three steps in the velocity calibration. First, the differences in the displacements between the InSAR-derived velocity map and our calculated ice velocity maps from Landsat images are calculated in the stagnant areas. Second, to eliminate outliers, a 3σ filter is recursively applied to identify differences. In this technique, the measurements are removed if the magnitudes of the values are larger than three times the standard deviation (3σ). Third, the mean of the remaining differences is considered the offset of the displacement scenes. Furthermore, the offsets of the displacement

scenes outside of stagnant areas (such as in the Ross and Ronne ice shelves) are estimated by overlapping neighbouring scenes captured at approximately the same time. The offsets of two velocity components are independently estimated. In addition, for computational efficiency, Antarctica is divided into 11 sub-regions, and data stacking is independently performed. Finally, the 11 sub-regions are mosaicked to generate an ice velocity map of all of Antarctica.

The mosaicked velocity maps are produced based on the displacement scenes. To increase the accuracy of the mosaicked velocity maps, we stack all displacement scenes after removing the pixels with an SNR less than 0.9. In general, a velocity map contains 8–10 scenes in a given location. For a specific pixel denoted as *i*, all displacement scenes (m=1, 2, …, n) are stacked to obtain the estimate of the ice velocity (V_i) as follows:

$$V_i = \frac{\sum_{m=1}^{n} \Delta d_m^i}{\sum_{m=1}^{n} \Delta t_m^i} \qquad (1)$$

where Δd_m^i denotes the generated displacement during the given time interval Δt_m^i.

7 Antarctic-wide ice velocity estimates

Due to the small number of images available during the time period of our survey, it is difficult to produce an individual mosaic for all of Antarctica in 2013 and 2016. Thus, the images acquired within two years are used to produce the 2014 mosaic and the 2015 mosaic. In Antarctica, the valuable L8 images are only available for the summer and fall seasons, i.e., in November, December, January, February and March, which means

that the L8 ice velocities represent the summer/fall ice velocity. In Supplementary Figure 1, we show mosaicked ice velocity maps for 2014 (Supplementary Fig. 1a) and 2015 (Supplementary Fig. 1b) over all of Antarctica. Ice velocity differences between the two maps are usually very small relative to the magnitudes of the velocities because of the relatively small mean (0.17 m yr^{-1}) and standard deviation (7.6 m yr^{-1}) (Supplementary Fig. 1d). The InSAR-derived ice velocity data[64] from approximately 2008 are also shown (Supplementary Fig. 1c). The data at grounding lines are used as references to estimate glacier discharge changes from ~2008 to 2014 and 2015. Our velocity result depicts an ice flow field pattern similar to those of the InSAR-derived ice velocity data[65] and the recently released annual InSAR-derived ice velocity maps[64]. The spatial resolution of our ice velocity data is 100 m, which is 4 to 10 times finer or higher than that of the InSAR-derived ice flow maps[64,65]. Our two ice velocity maps thus provide the first opportunity to investigate localized ice dynamics, such as crevasse formation, and the roles of ice rises and rumples in ice-sheet dynamics and evolution. These maps also have good coverage over Antarctica, except south of 82.5° S. The two mosaicked ice velocity maps cover the majority of the Antarctic ice sheet and nearly 99% of the fast-flowing glaciers and ice shelves, as well as fast ice, except for a few ice streams located on the Ronne Ice Shelf (e.g., Academy and Foundation glaciers) and the Ross Ice Shelf (e.g., Whillans glacier on the Siple Coast).

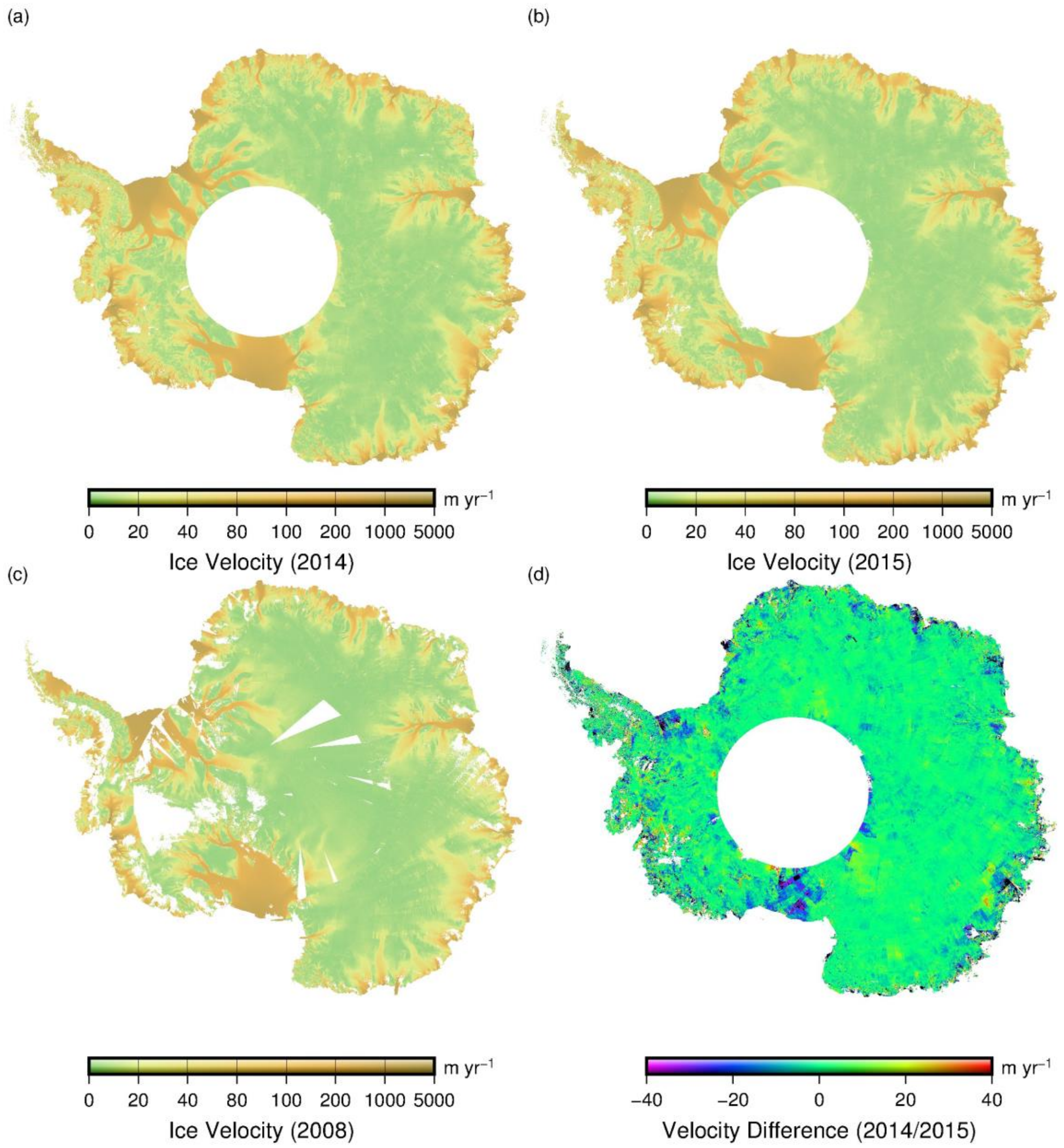

Supplementary Figure 1. (**a**), L8-derived '2014' ice velocity estimates from December 2013 to December 2014; (**b**), L8-derived '2015' ice velocity estimates from January 2015 to March 2016; (**c**), InSAR-derived '2008' ice velocity estimates[64] from 2007 to 2009; and (**d**), the difference in ice velocity estimates between '2015' and '2014'. The L8-derived ice velocity maps are drawn on a 500-m grid. The maps were created using The Generic Mapping Tools version 5.2.1 (http://gmt.soest.hawaii.edu/).[82]

8 Uncertainty analysis

8.1 Uncertainty of ice velocity maps

The uncertainty in the ice velocity maps derived from the L8 data primarily results from co-registration accuracy, the time interval between the pairs used to extract displacement vectors, and the amount of stacking data. The co-registration accuracy is mainly dependent on three main error sources: (1) decorrelation due to dramatic ground changes, a lack of measurable features between scenes due to long time intervals or low-contrast land cover (e.g., snow or ice); (2) low image quality caused by sensor noise, pixel oversaturation, aliasing and cloud contamination; and (3) topographic artefacts caused by shadowing differences and the inaccurate orthorectification of satellite attitudes. It is difficult to quantify the respective effects of the three error sources; here, the co-registration accuracy is conservatively set to 1/10 of the pixel size for the E-W and N-S displacement components. This value is larger than the value of 1/50 the pixel size proposed by Leprince et al. (2007)[80]. Using the co-registration error, together with the total quantity of stacking data and the time interval between two acquisitions, the ice velocity error can be estimated based on the method of error propagation.

According to the mosaicking method discussed above (Eq. 1), the uncertainty in one mosaicked velocity component for the i-th pixel (denoted by $\sigma_{\mathrm{V_i}}$) can be estimated using the following error propagation formula under the assumption that the errors from different sources are independent:

$$\sigma_{\mathrm{V_i}} = \pm\sqrt{\sum_{m=1}^{n}(\sigma_{\mathrm{m}}^{\mathrm{i}})^2 \Big/ \left(\sum_{m=1}^{n}\Delta t_{\mathrm{m}}^{\mathrm{i}}\right)^2} \tag{2}$$

where $\sigma_{\mathrm{m}}^{\mathrm{i}}$ is the co-registration error, i.e., the standard deviation of the m-th displacement observation during the time interval $\Delta t_{\mathrm{m}}^{\mathrm{i}}$. Since the co-registration errors

are constant in the spatial (the whole scene) and temporal domains (all stacked displacements), if $\sigma_{\mathrm{m}}^{\mathrm{i}}$ is assumed to be a constant of σ, Equation (2) can be simplified as follows.

$$\sigma_{\mathrm{V_i}} = \pm\sqrt{\mathrm{n}}\sigma / \sum_{m=1}^{n} \Delta t_{\mathrm{m}}^{\mathrm{i}} \tag{3}$$

The uncertainty in a mosaicked velocity map is dependent on the quantity of stacking data and the time intervals used during velocity stacking. Therefore, large time spans result in high ice velocity accuracy. Since the E-W and N-S components of the *i*-th pixel have the same uncertainty, which can be calculated with Equation (3), the uncertainty is valid for the magnitude of the velocity vector. The error in the magnitude of the mosaicked velocity vector for magnitudes between 0 and 20 m yr^{-1} is shown in Supplementary Figure 2a. For comparison, the uncertainty of the InSAR ice velocity estimates is shown in Supplementary Figure 2b.

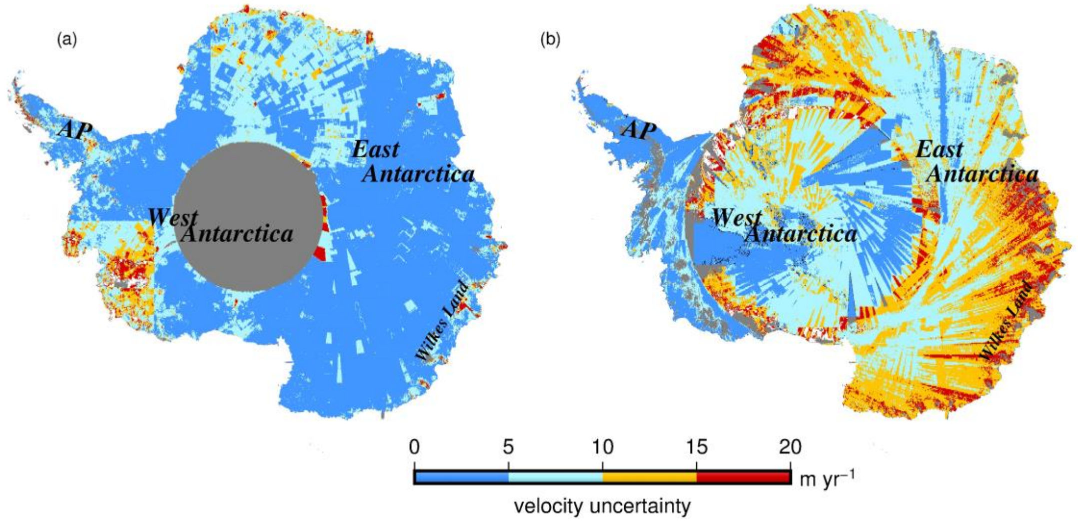

Supplementary Figure 2. Uncertainty maps of the L8-derived Antarctic ice velocity in 2015 (**a**) and InSAR-derived ice velocity[64] (**b**). The maps were created using The Generic Mapping Tools version 5.2.1 (http://gmt.soest.hawaii.edu/).[82]

Our ice velocity results are only compared with the *in situ* measurements located in the slow-flowing areas (<100 m yr^{-1}). The 314 sites chosen for the comparison are shown with dots in Supplementary Figure 3, and the colours of the dots denote the magnitudes of the differences. The upper inset shows the histogram of the differences between our velocity data and the *in situ* measurements. The differences are generally between –10 m yr^{-1} and 10 m yr^{-1}, and the mean and standard deviation are –2.4 m yr^{-1} and 8 m yr^{-1}, respectively. The non-zero mean of our results is likely attributable to the glacier deceleration along the Siple Coast of West Antarctica. For comparison, the differences between the InSAR velocity and field-surveyed data are shown in the lower inset in Supplementary Figure 3. The mean value of the differences is 0.3 m yr^{-1}, with a standard deviation of 12 m yr^{-1}.

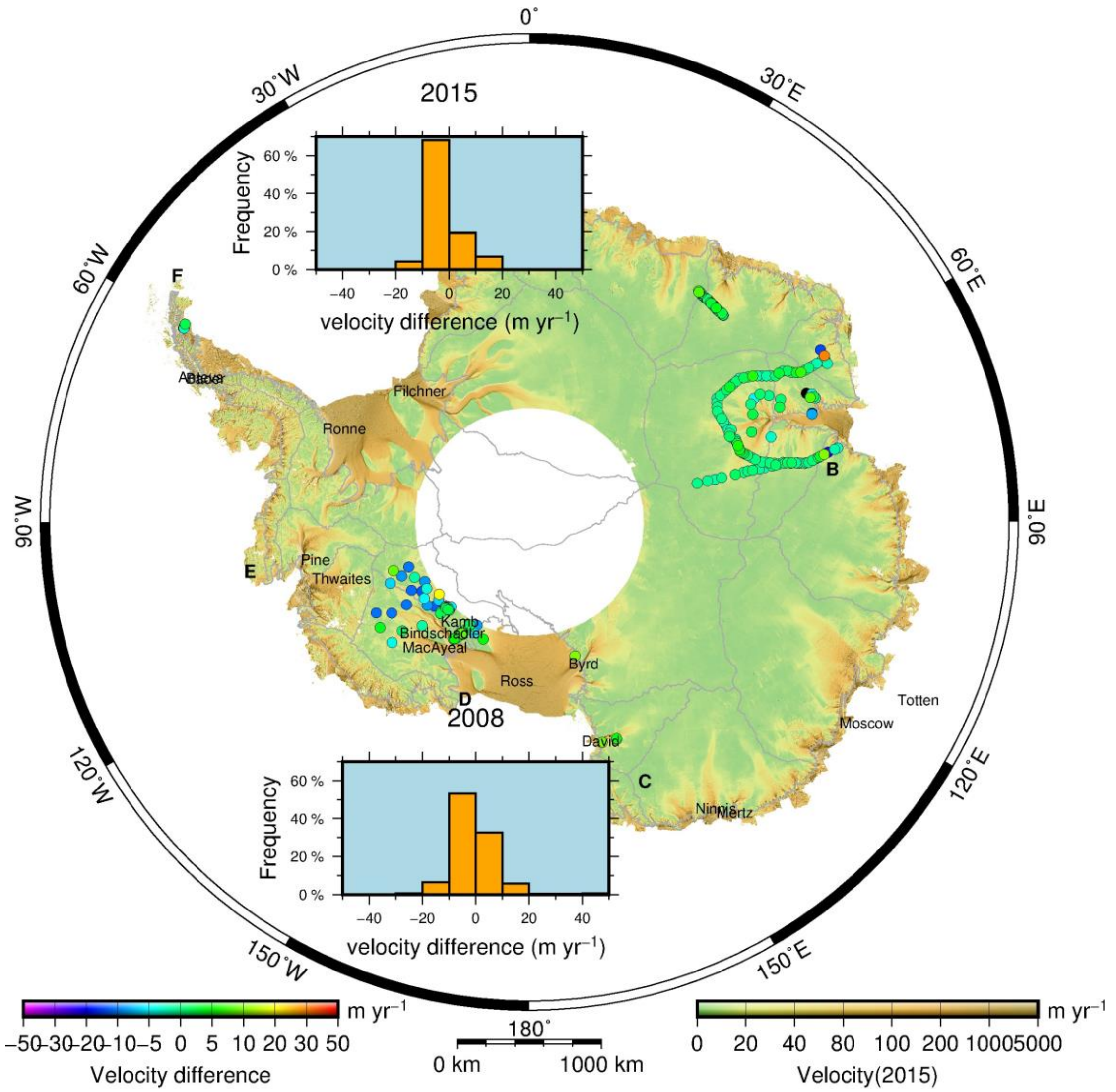

Supplementary Figure 3. The comparison between L8 ice velocities in 2015 and data from *in situ* measurements. The coloured dots show the differences between the L8-derived ice velocity in 2015 and *in situ* survey data. The upper inset shows a histogram of the differences between L8-derived ice velocity and field data, and the lower inset shows a histogram of the differences between InSAR ice velocity and field data. The solid grey lines delineate the major ice basins denoted by Zwally et al. (2012). The map was created using The Generic Mapping Tools version 5.2.1 (http://gmt.soest.hawaii.edu/).[82]

8.2 Uncertainty in the SMB

For each surface mass balance (SMB) grid in Antarctica (excluding ice shelves), we use the trend and coefficients associated with seasonal periodicities to fit the monthly time series of the accumulated SMB data using the least squares method. The standard deviation of the trend can be treated as one estimate of the uncertainty in the SMB. The grid uncertainties are simply summed to determine the uncertainties for 27 glacier basins. The total uncertainty of the entire Antarctic ice sheet is calculated using the uncertainty estimates in individual basins based on the method of error propagation. For the six oceanic sectors, the uncertainties in the corresponding glacier basins are summed to give the SMB uncertainty estimate. All the SMB uncertainties can be found in Tables 1 and S2. The total uncertainty of the SMB for the entire Antarctic ice sheet is ±58 Gt yr^{-1}.

8.3 Uncertainty of ice discharge

8.3.1 Method

In the process of calculating ice discharge across a grounding line, the grounding line is divided into *K* segments; thus, the uncertainty in the ice discharge (denoted σ_{GLF}) can be estimated using the following error propagation equation:

$$\sigma_{\mathrm{GLF}}^2 = \sum_{k=1}^{K} \Delta m_k^2 \left(\frac{\sigma_V}{V}\right)^2 + \sum_{k=1}^{K} \Delta m_k^2 \left(\frac{\sigma_T}{T}\right)^2 \tag{4}$$

where Δm_k is the ice discharge across the *k*-th segment; the two *k*-th terms together denote the variance contributions from the *k*-th segment; σ_V and σ_T denote the uncertainties in the ice velocity (V) and ice thickness (*T*) in the *k*-th segment

respectively; the parameter σ_V is defined in section 8.1; and σ_T is discussed in section 8.3.2.

8.3.2 Uncertainty in the ice thickness (σ_T)

The uncertainty of the ice thickness inferred from Cryosat-2 altimetry measurements will be analysed in detail in another paper (in preparation). Here, we show only the estimate using airborne IPR measurements of the Amery Ice Shelf[83]. The differences between CS2-derived ice shelf thickness and IPR measurements are calculated. More than 260,000 IPR measurements have been observed by the Australian National Antarctic Research Expedition (ANARE), and these values have a good thickness accuracy in the range of 30 to 75 m. Based on the processing method provided by Chuter et al. (2015), more than 92% of points are within 100 m, and the mean bias and standard deviation between the two independent data sets are –26 m and 37 m, respectively. This result suggests that our CS2-derived ice thickness data are more accurate.

For the uncertainties associated with the Bedmap2 ice thickness[84], if the thicknesses are measured by radar and seismic techniques, they are usually less than ±51.2 m, but for the thicknesses of ice shelves derived from altimetry data under the assumption of hydrostatic equilibrium, the uncertainties are usually between ±100 m and ±150 m. Thus, we do not use the ice thicknesses of ice shelves. The absolute errors of the IPR ice thickness data are ±44.5 m for MCorDS[76] and ±10 m for HiCARS[85].

9 Mass balance of the ice sheet

The SMB in Antarctica is dominated by precipitation (snowfall). Here, we calculate

the inflow mass (i.e., SMB) using a new SMB data product (1979–2014)[86] at a horizontal resolution of 27.5 km for Antarctica (excluding the Antarctic Peninsula). This product is generated using the regional atmospheric climate model RACMO2.3 and a firn densification model (FDM)[87] for each of the 27 glacier drainage basins[88] (Supplementary Fig. 4). For the Antarctic Peninsula, we use a high-resolution surface mass balance model over the time period of 1979–2014. This product is also generated using RACMO2.3 and the FDM[89]. In the Antarctic Peninsula, the RACMO2.3 hydrostatic model operates at a horizontal resolution of ~5.5 km and includes 40 vertical levels. These specifications are suitable for the peninsula due to its sharp climatic gradients and steep mountainous terrain[90]. The model combines the dynamics package of the High Resolution Limited Area Model (HIRLAM) with the physics package of the European Centre for Medium-range Weather Forecasts (ECMWF) Integrated Forecast System (IFS) (ECMWF-IFS, 2008)[86].

The SMB values are estimated for the 27 glacier drainage basins (Table S3). The total SMB of the Antarctic ice sheet is 1,901 Gt yr^{-1}, with an interannual variability of 58 Gt yr^{-1}, which is similar to a newly published result using the same SMB product[86] and comparable to the result of a previous study of 1,983±122 Gt yr^{-1}.[91] In the Antarctic Peninsula (AP), West Antarctica (WA) and East Antarctica (EA), SMB values account for 12%, 30% and 58%, respectively, of the total value for the entire Antarctic ice sheet, and the snow accumulation rates per unit area are 939 mm yr^{-1}, 330 mm yr^{-1}, and 111 mm yr^{-1}, respectively. The average accumulation rate over the entire ice sheet is 160 mm yr^{-1}.

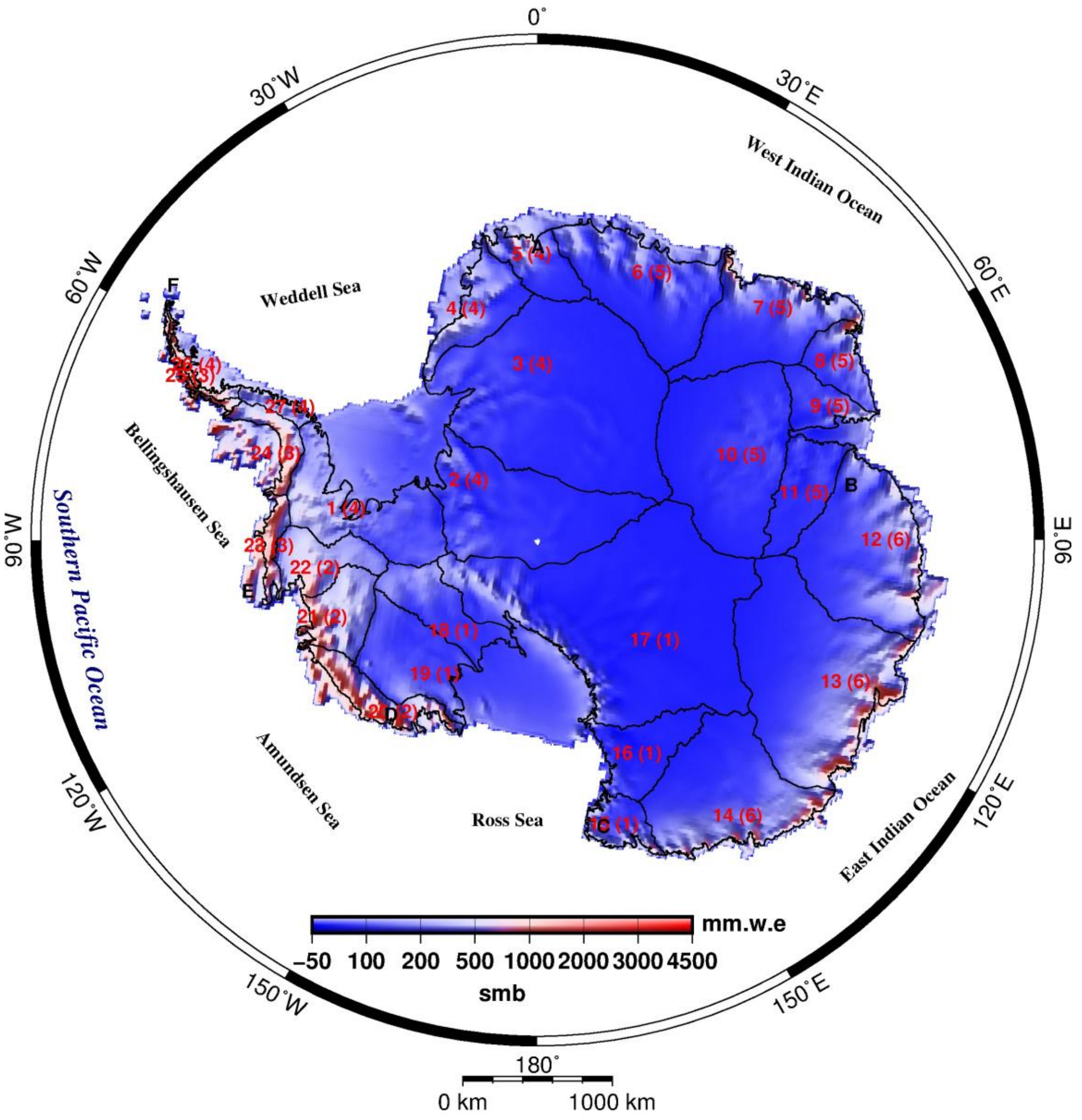

Supplementary Figure 4. The SMB, an average of snow accumulation from 1979 to 2014 simulated by the RACMO2.3[86] and FDM models[87]. The magnitude of the SMB is colour-coded (in mm yr^{-1} water equivalent). The grounded ice sheets are labelled based on the major basins (divided by solid black lines). Basins and the affiliated oceanic sectors are denoted by numbers and the accompanying numbers in parentheses, respectively: 1. Ross Sea (ROS); 2. Amundsen Sea (AMU); 3. Bellingshausen Sea (BEL); 4. Weddell Sea (WED); 5. West Indian Ocean (WIS); and 6. East Indian Ocean

(EIS). The map was created using The Generic Mapping Tools version 5.2.1 (http://gmt.soest.hawaii.edu/).[82]

The mass discharge across the grounding line is calculated with the rigorous flux gate method[60] in combination with the ice velocity and a compilation of Cryosat2 ice thickness and Bedmap2 ice thickness data associated with IPR track measurements between 2002 and 2014 from the Operation IceBridge (OIB) project (Table S2). Our procedure integrates the ice flux estimates along the grounding line pixel-by-pixel considering the directions of ice velocity vectors and the orientation of the grounding line. This approach can minimize the influence of geolocation errors of grounding lines. The total mass discharges across the grounding lines in Antarctica were estimated to be 2,050±41 Gt yr^{-1}, 2,141±42 Gt yr^{-1} and 2,131±42 Gt yr^{-1} in 2008, 2014 and 2015, respectively. Our estimate for 2008 is similar to recently published estimates[59,60]. Note that the mass discharge in the BEL sector is likely underestimated because the mass discharge associated with summertime surface meltwater (related to high air temperatures above the freezing point) is not considered. Negative ice flux estimates are obtained for several small glaciers and are likely due to grounding line geolocation errors or uncertainty in the ice velocity estimates. These error sources have no effect on the resulting mass balance estimates for Antarctica as a whole.

10 Glacier dynamics

10.1 Ross Sea

Glaciers related to the Ross Ice Shelf. Along the Siple Coast (Supplementary Fig.

5), the Bindschadler glacier (formerly called Ice Stream D) decelerated by 7% between 2015 and 2008, and the MacAyeal ice stream exhibited no significant change. Note that our measurements do not cover the Kamb, Whillans, and Van der Venn glaciers along the Siple Coast. The mass discharge along the Siple Coast over the past seven years shows no significant change, remaining at 0.3 Gt yr^{-1}. In the Transantarctic Mountains, the Marsh glacier shows no significant change; however, the largest glacier, the Byrd glacier, shows a deceleration of approximately 7% from 2008 to 2015, as compared with 15% based on earlier measurements in 1978[72,92]. To the north of the Byrd glacier, the Mulock glacier shows no significant change. The mass discharge in the Transantarctic Mountains (basin 17) also shows no obvious change from 2008 to 2015.

Glaciers in Victoria Land. The glaciers in Victoria Land show apparently contrasting ice dynamic behaviours. The small glaciers exhibit widespread accelerations. Large accelerations are observed for the David glacier (24%), Irving glacier (23%), and Tucker glacier (23%) over the past seven years. However, the Amos glacier exhibits obvious deceleration (–35%). In addition, some glaciers, such as the Aviator, Borchgrevink, Mariner, and some small unnamed glaciers, show no significant change. The distinct contrasting glacier behaviours in Victoria Land lead to no significant mass change in the area.

Glaciers in George V Land. The majority of the glaciers in George V Land exhibit accelerations of 17% to 224%. The large Rennick glacier displays a deceleration of –15%, the small Crume and O'Hara glaciers exhibit accelerations of 31% and 224%, respectively. The Noll glacier and an unnamed glacier accelerate by 27% and 17%,

respectively.

The apparent differences in ice dynamics in Victoria Land (basins 15 and 16) and George V Land (basin 15), compared with those for the Ross ice streams, may be caused by warmer Circumpolar Deep Water (CDW), which has been intruding cavities along the grounding lines[93]. In contrast, the Ross ice streams are not affected by the CDW and feature relatively colder subglacial environments due to low temperature ice shelf meltwater from the larger Ross Ice Shelf.

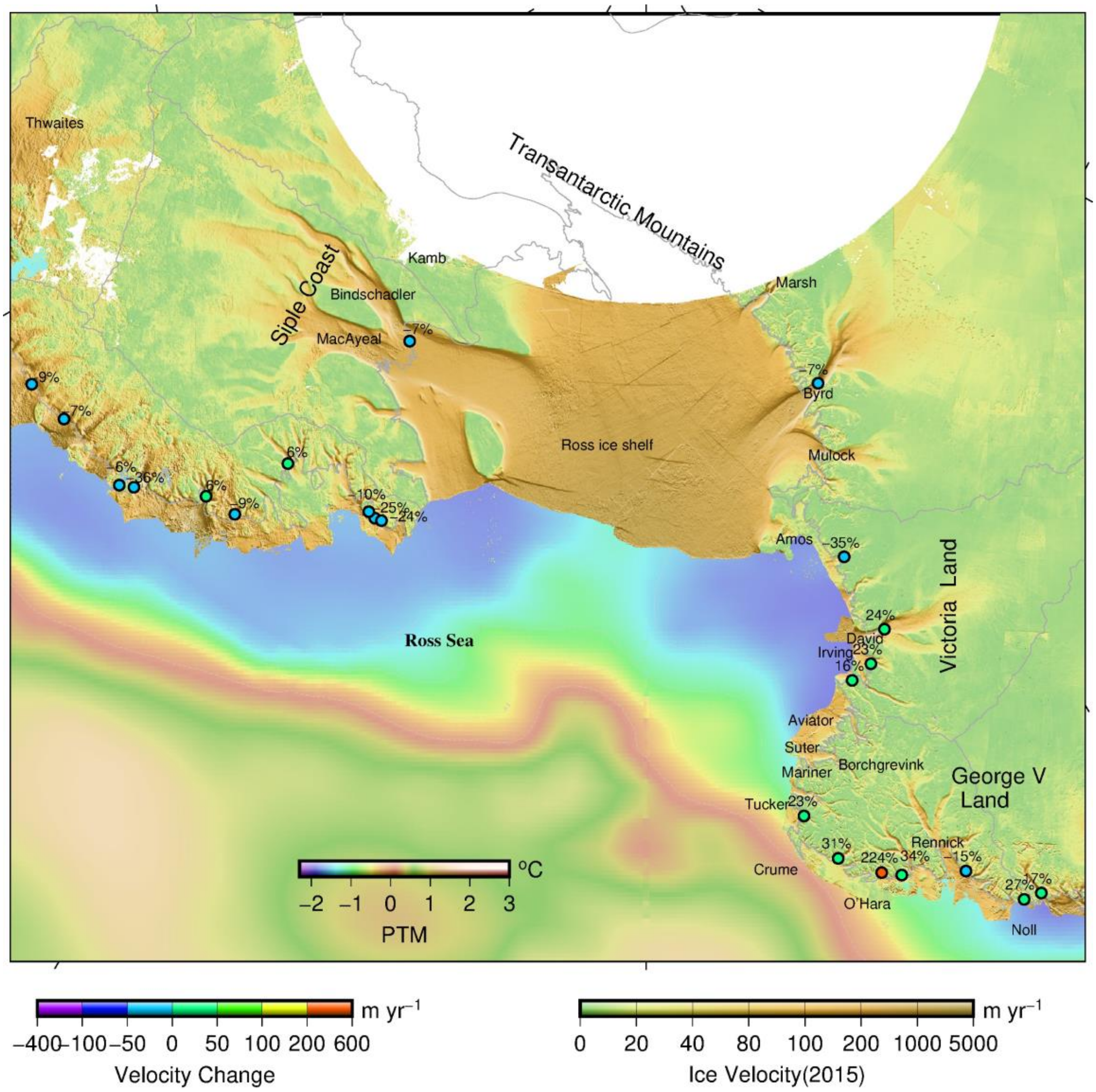

Supplementary Figure 5. The ice velocity changes between 2015 and 2008 in the Ross Sea sector. The coloured dots show the magnitudes of velocity changes, and the

numbers represent percentage differences in velocities between 2008 and. The locations and glaciers noted in the text are labelled. The ice velocity is colour coded on a logarithmic scale and overlaid on gridded potential seawater temperature data (PTM) at 200-m depth from the World Ocean Circulation Experiment (WOCE). Grey lines delineate the glacier basins and grounding lines. The map was created using The Generic Mapping Tools version 5.2.1 (http://gmt.soest.hawaii.edu/).[82]

10.2 Amundsen Sea

One of the three Glaciers draining into the Sulzberger Ice Shelf (Supplementary Fig. 6) has accelerated by 6% and directly led to an accelerated mass discharge of 3.3 Gt yr^{-1} between 2008 and 2015. The Land glacier exhibits an acceleration of 6%. However, the tributary glaciers of the Getz Ice Shelf, the largest ice shelf in the Amundsen Sea, show significant decelerations from 6% to 16%, although one glacier accelerates by 7%. These decelerations correspond to a clear deceleration in ice discharge (2.2 Gt yr^{-1}) from 2008 to 2015. The Kohler glacier of the Dotson Ice Shelf exhibits a deceleration of 17%, and the Crosson glacier displays a deceleration of 8%. However, the Pine Island and Thwaites glaciers accelerate by 1% and 8%, respectively. The speedup in the Pine Island glacier led to an increased mass discharge of 1.3 Gt yr^{-1} towards the Pine Island Ice Shelf between 2008 and 2015. The Thwaites glacier speedup also contributed to a significant increase in mass discharge of 14 Gt yr^{-1}. The rapid mass discharge in the Pine Island and Thwaites catchments cannot be fully explained by warmer atmospheric conditions. The oceanic thermocline conditions may be one of the main factors that resulted in the differences in ice dynamics, as warmer CDW intrusions have been

confirmed by conductivity-temperature-depth (CTD) observations[94,95]. Additionally, rapid basal melting and thinning in adjacent ice shelves are likely the other main factors that have led to ice flow acceleration[94], as these processes partially reduce or eliminate the buttressing force of the ice shelves[96]. We estimate a mass discharge increase of 14±4 Gt yr^{-1} over the past seven years, i.e., 2008–2015, which is lower than the value of 46±5 Gt yr^{-1} estimated for the previous decade, i.e., 1996–2006[97].

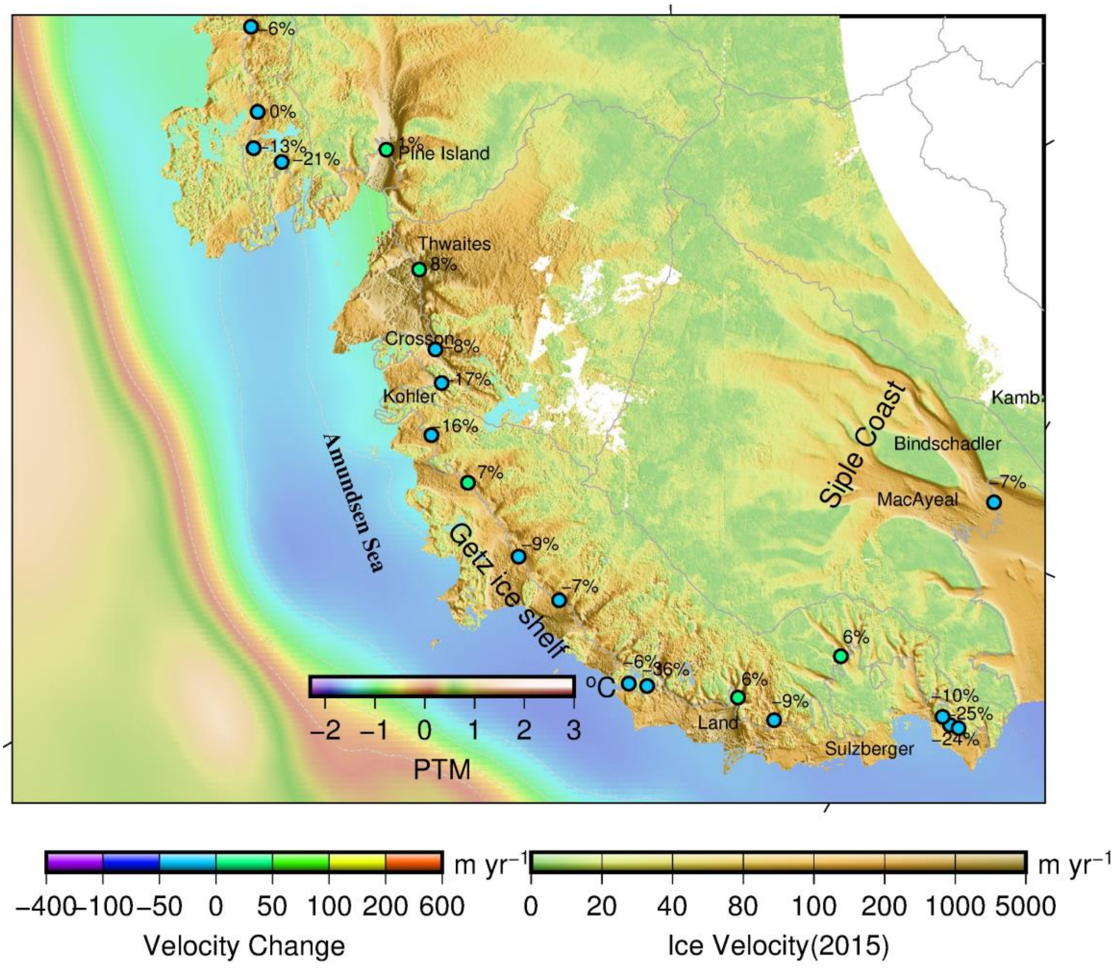

Supplementary Figure 6. Same as Supplementary Figure 5 but in the Amundsen Sea sector. The map was created using The Generic Mapping Tools version 5.2.1 (http://gmt.soest.hawaii.edu/).[82]

Multiple ice velocity observations since 1996 are available for the Pine Island glacier (Supplementary Fig. 7), the Thwaites glacier (Supplementary Fig. 8), and the associated ice shelves[98]. These observations allow us to investigate the ice dynamics in this region over a long time period. Here, we investigate the profiles along the ice flow centre line from the upstream ice streams to the ice shelves. Both the Pine Island glacier and its ice shelf exhibit acceleration patterns with greater acceleration from 1996–2006 than from 2008–2015. The slowdown in acceleration may indicate the cessation of an episode of fast retreat or a short-lived decrease in acceleration[99]. The current dynamics of the glacier seem to be controlled by the ice-shelf dynamics based on the synchronous changes in the glacier and ice shelf and the increasing meltwater production beneath the ice shelf [95,100]. The Thwaites glacier exhibits similar ice dynamics, but the temporal pattern of ice velocity is distinctly different. The velocities of the glacier and ice shelf suddenly increase in 2005 then remain stable, and the acceleration seems to affect only the area 20 km upstream of the grounding line. The significant difference between the upstream and downstream areas of the glacier is likely attributable to the bathymetry, which features a relative high ridge 20 km upstream of the grounding line. Hence, the warmer CDW has likely not yet invaded the area upstream of the ridge. However, in the Pine Island glacier, acceleration in the area 200 km upstream of the grounding line can be observed. This acceleration is caused by a seaward-sloping bed below the upstream portion of the glacier, and the warmer CDW has likely intruded into the inland sloping bed in the vicinity of the grounding line. The different bathymetric configurations likely lead to distinct dynamic patterns in the two adjacent glaciers,

although both are subjected to the same atmospheric and oceanic forcing.

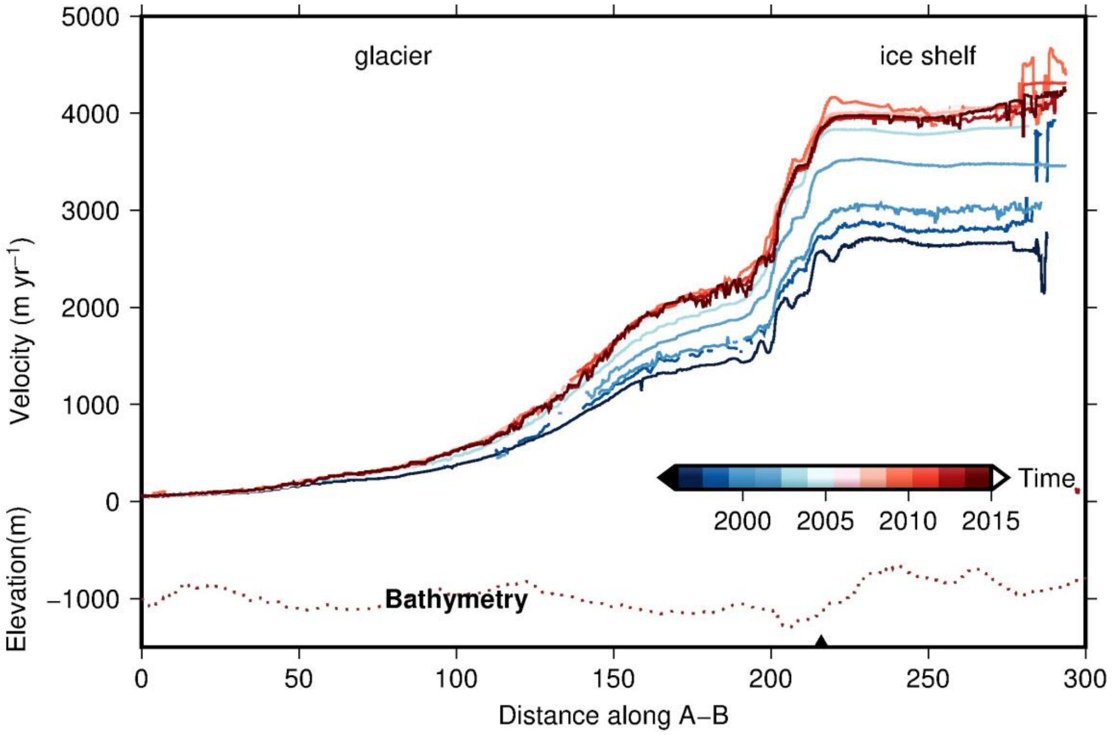

Supplementary Figure 7. Ice dynamics of the Pine Island glacier and its ice shelf. The dotted line shows the bathymetry, and the black triangle indicates the location of the grounding line. The ice velocity curves in the entire survey period are colour coded based on acquisition dates. The map was created using The Generic Mapping Tools version 5.2.1 (http://gmt.soest.hawaii.edu/).[82]

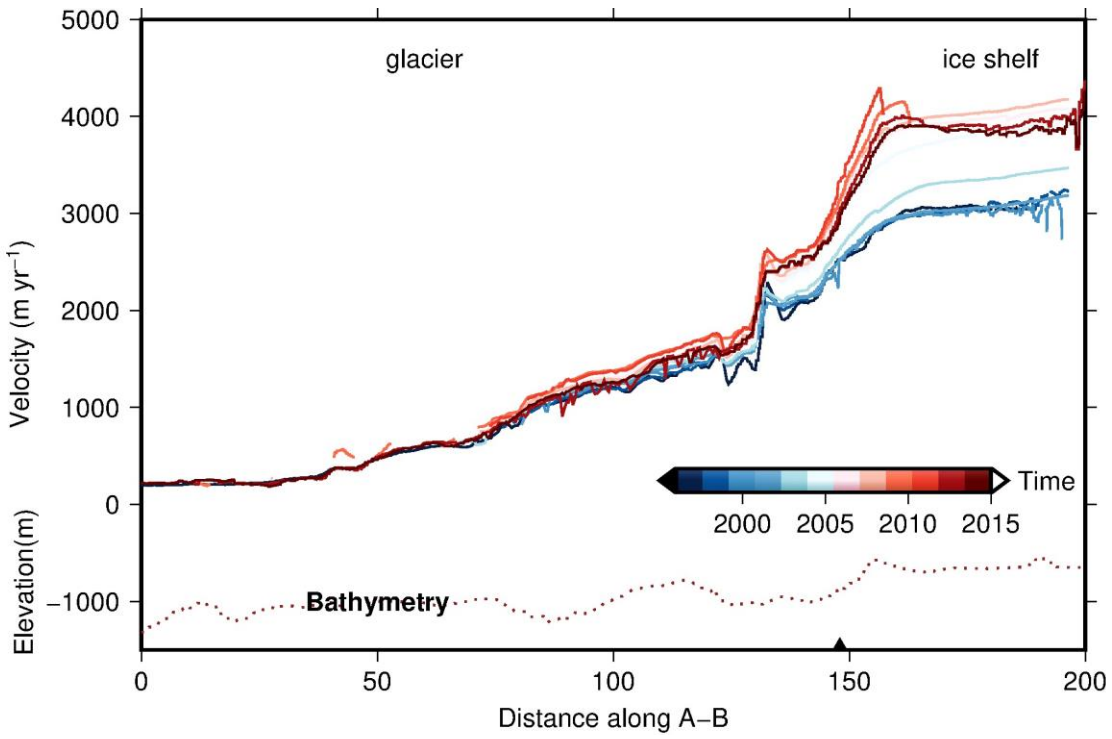

Supplementary Figure 8. Same as Supplementary Figure 7 but for the Thwaites glacier and its ice shelf. The map was created using The Generic Mapping Tools version 5.2.1 (http://gmt.soest.hawaii.edu/).[82]

10.3 Bellingshausen Sea

In the Antarctic Peninsula, the largest accelerations (>50%) occurs for small glaciers and results in an accelerated mass discharge of 11±6 Gt yr^{-1}. We find that the InSAR-derived ice velocities (shown as white dots in Supplementary Fig. 9) are unrealistically low in the grounding line zone in the northwest AP. The accelerated mass discharge is 2±3 Gt yr^{-1} if the glaciers in the northwest AP (basin 25) are assumed to have undergone no change between 2008 and 2015. The accelerated mass discharge is likely attributable to the intrusion of warmer CDW into the cavities in these glaciers and the significant increase in air temperature in the AP[101-105]. The velocity of the Prospect glacier, which

feeds into the Wordie Ice Shelf, increased by 26% from 2008 to 2015. This glacier and two unnamed glaciers that feed into the ice shelf contribute to the largest total increases in mass discharge (~9 Gt yr^{-1}) in the Antarctic Peninsula. However, the glaciers draining into the George VI Ice Shelf exhibit no significant increases in mass discharge. Furthermore, the glaciers draining into the large Abbot and Venable ice shelves clearly display decelerated mass discharges of 4 Gt yr^{-1} and 1 Gt yr^{-1}, respectively.

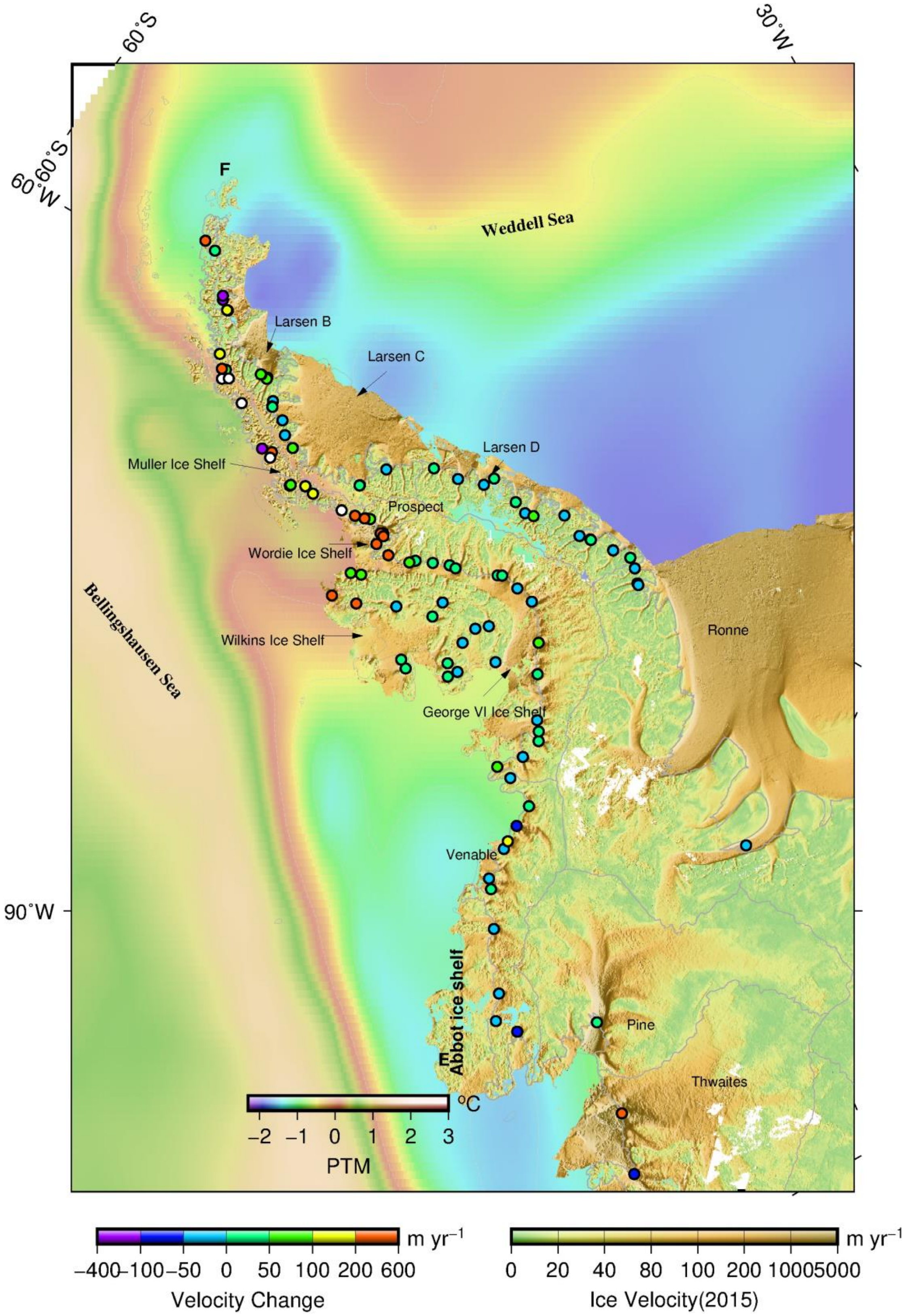

Supplementary Figure 9. Same as Supplementary Figure 5 but for the Antarctic Peninsula. The white dots indicate that the changes are larger than 600 m yr^{-1}. The map was created using The Generic Mapping Tools version 5.2.1

(http://gmt.soest.hawaii.edu/).[82]

10.4 Weddell Sea

The Ronne-Filchner catchment (Supplementary Fig. 10) exhibits no significant mass change (8±30 Gt yr^{-1}). The majority of the glaciers show no significant change. In the Ronne Ice Shelf, only Rutford and two unnamed glaciers display apparent decelerations ranging from 9% to 22%. In the Filchner Ice Shelf, only Bailey glacier exhibits a significant deceleration of 17%. Moreover, in the Filchner catchment, there is no significant change in mass discharge (0.6±5 Gt yr^{-1}).

In the Brunt and Riiser catchments (basin 4), a slightly accelerated mass discharge (6±6 Gt yr^{-1}) is caused by the acceleration of some small glaciers (Supplementary Fig. 10, Table S2). For example, the velocities of the Quar glacier and two unnamed glaciers have accelerated by 26%, 34% and 11%, respectively. However, the Stancomb-Wills glacier flowing into the Brunt Ice Shelf displayed no significant change over the past seven years. The Veststraumen glacier draining into the Riiser Ice Shelf also exhibits no significant change. The widespread decelerated flow of glaciers draining into the Ekstrom, Atka, Jelbart and Fimbul ice shelves in basin 5 has directly led to a total mass gain of nearly 5 Gt yr^{-1} over the past seven years. However, only Balakirev glacier exhibit a significant deceleration of 53%, and other glaciers show no apparent changes.

In the eastern Antarctic Peninsula (EAP) (see Supplementary Fig. 9), the mass discharge of the glaciers in the Larsen B Ice Shelf has increased by 20% since 2008. The glaciers in the Larsen B Ice Shelf catchment accelerated by 10–16%. The fast

motion and accelerated flows observed in the Larsen B glacier may be linked to the abrupt disintegration of the Larsen B Ice Shelf in March 2002, as the loss of the ice shelf would have removed the buttressing force[104,106]. Another region with a rapid accelerated discharge of up to 100% is the catchment surrounding the Larsen A Ice Shelf, which broke off in 1995. The majority of other glaciers in the EAP also display complicated patterns. Some glaciers exhibit acceleration, and other glaciers decelerate. Compared with the EAP, the acceleration of the glaciers in the WAP is more significant and predominately caused by an oceanic driving mechanism[107] and warmer air temperatures[105]. In contrast, the east coast is governed by cold oceanic conditions in the Weddell Sea and the obstacles formed by high mountains. The glaciers in the Weddell Sea sector situated in East Antarctica display dramatic differences in ice dynamics.

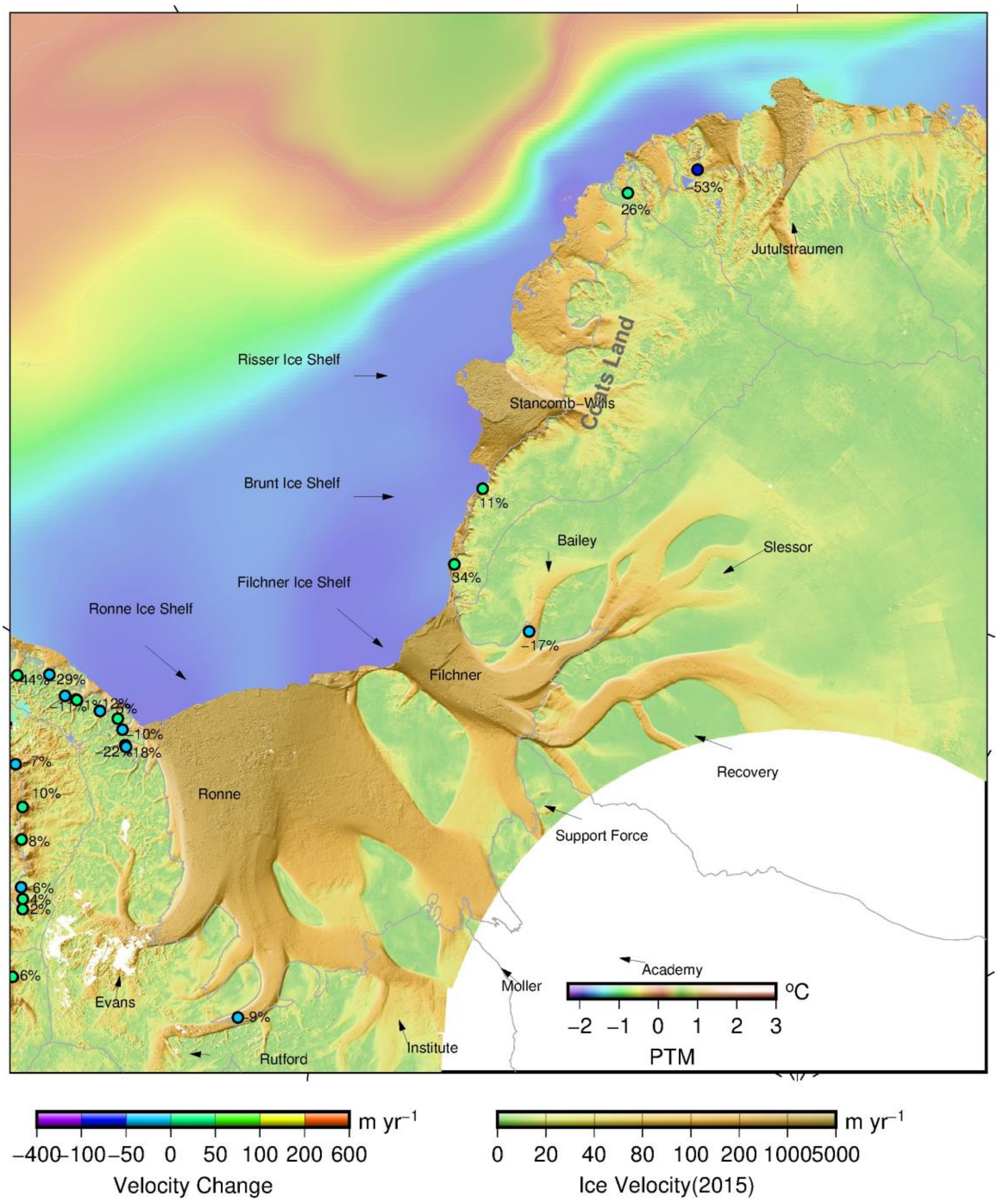

Supplementary Figure 10. Same as Supplementary Figure 5 but for the Weddell Sea. The map was created using The Generic Mapping Tools version 5.2.1 (http://gmt.soest.hawaii.edu/).[82]

10.5 West Indian Ocean

Increased mass discharges (basin 7) in Enderby Land, are mainly caused by the

speedup of the Rayner glaciers toward Casey Bay, glaciers adjacent to the Syowa (Japan) scientific expedition station, and some glaciers near Lutzow-Holm Bay (Supplementary Fig. 11). Slightly decelerated mass discharge in basin 8 is caused by the deceleration of the Robert, Wilma, and Edward glaciers towards Edward VIII Bay. In Dronning Maud Land, to the west of Enderby Land, the discharges of many glaciers decelerated from 2008 to 2015. For example, the mass discharges of the Shirase and Jutulstraumen glaciers decreased by 2%. In the Amery system, the three largest glaciers, the Lambert, Mellor and Fisher glaciers, also exhibit no significant ice velocity changes.

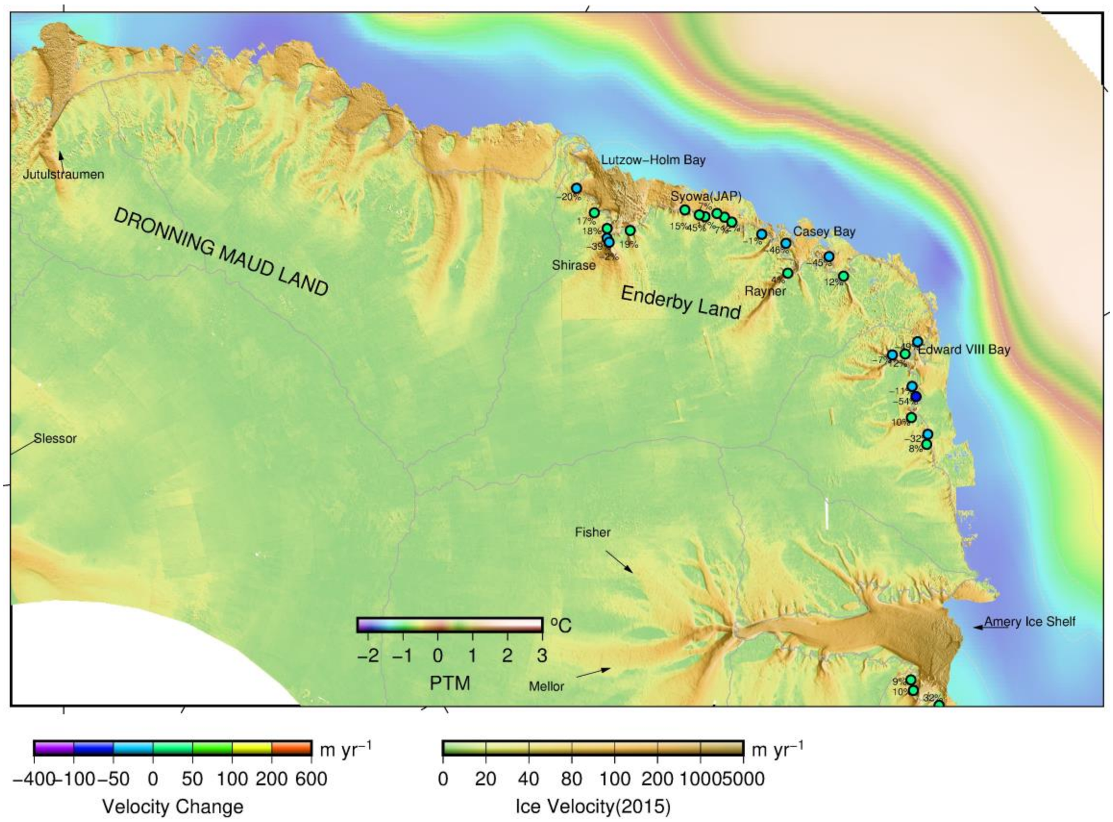

Supplementary Figure 11. Same as Supplementary Figure 5 but for the West Indian Ocean. The map was created using The Generic Mapping Tools version 5.2.1 (http://gmt.soest.hawaii.edu/).[82]

10.6 East Indian Ocean

In Wilkes Land, accelerated mass discharges are observed in all basins in the sector (Supplementary Fig. 12). The accelerated mass discharges are 13±6 Gt yr^{-1} in basin 12, 18±11 Gt yr^{-1} in basin 13, and 19±4 Gt yr^{-1} in basin 14. The increased mass losses in these glacier basins are associated with bathymetric configurations similar to that of the West Antarctic ice sheet[108], a newly found trough beneath the Totten glacier[85,109] (Fig. 3), and the potential CDW intrusion[110,111]. These factors may eventually lead to the destabilization of the low-lying marine region, which would increase the instability risk in the marine-based sector of East Antarctica in the future.

In ASB (basin 13), similar to the results of a previous study[112], the Totten glacier has accelerated by 4%, but the Moscow glacier exhibits no significant change. The difference likely results from local oceanic conditions, such as thickening in Moscow and thinning in Totten driven by ocean-forced instabilities[113]. The Frost glacier has also accelerated by ~8%.

The WSB (basin 14), which holds an ice mass more than five times that of West Antarctica[108], also exhibits an evident accelerated mass discharge (19 Gt yr^{-1}), which is consistent with a recently published study based on satellite altimetry measurements[114]. The majority of the glaciers in the area, such as the Ninnis, Mertz, Dibble, and Cook glaciers, have accelerated.

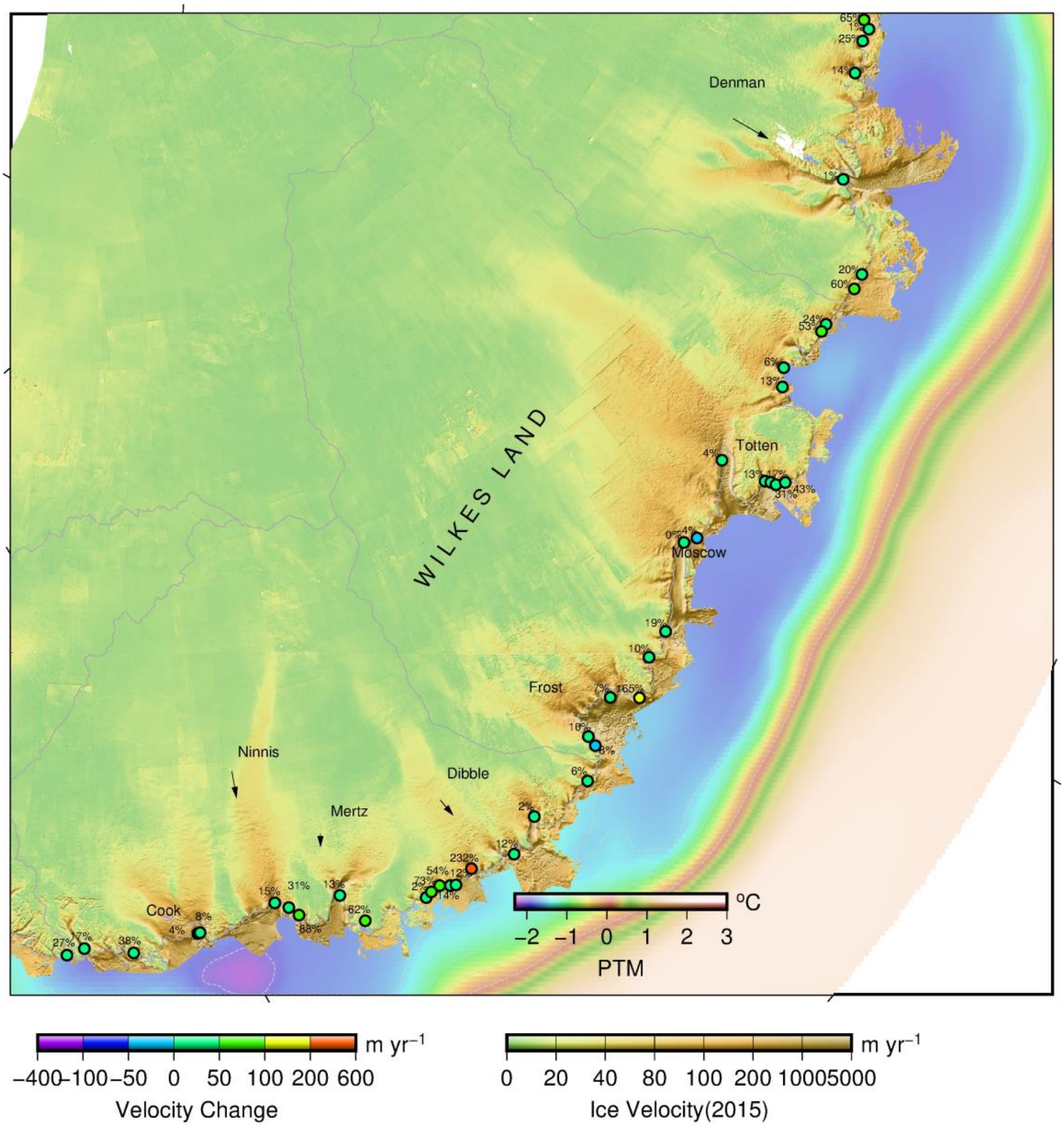

Supplementary Figure 12. Same as Supplementary Figure 5 but for the East Indian Ocean. The map was created using The Generic Mapping Tools version 5.2.1 (http://gmt.soest.hawaii.edu/).[82]

References

59 Depoorter, M. A. *et al.* Calving fluxes and basal melt rates of Antarctic ice shelves. *Nature* **502**, 89-92 (2013).

60 Rignot, E., Jacobs, S., Mouginot, J. & Scheuchl, B. Ice Shelf Melting Around Antarctica. *Science* **341**, 266-270 (2013).

61 Fahnestock, M. *et al.* Rapid large-area mapping of ice flow using Landsat 8. *Remote Sensing of Environment* **185**, 84-94 (2016).

62 Morfitt, R. *et al.* Landsat-8 Operational Land Imager (OLI) Radiometric Performance On-Orbit. *Remote Sens-Basel* **7**, 2208-2237 (2015).

63 Zanter, K. Landsat 8 (L8) Data users handbook, https://landsat.usgs.gov/landsat-8-l8-data-users-handbook (2016).

64 Mouginot, J., Rignot, E., Scheuchl, B. & Millan, R. Comprehensive Annual Ice Sheet Velocity Mapping Using Landsat-8, Sentinel-1, and RADARSAT-2 Data. *Remote Sens-Basel* **9**, 364 (2017).

65 Rignot, E., Mouginot, J. & Scheuchl, B. Ice flow of the Antarctic ice sheet. *Science* **333**, 1427-1430 (2011).

66 Rott, H., Rack, W., Nagler, T. & Skvarca, P. Climatically induced retreat and collapse of northern Larsen Ice Shelf, Antarctic Peninsula. *Ann. Glaciol* **27**, 86-92 (1998).

67 Skvarca, P., Rack, W. & Rott, H. 34 year satellite time series to monitor characteristics, extent and dynamics of Larsen B Ice Shelf, Antarctic Peninsula. *Ann. Glaciol* **29**, 255-260 (1999).

68 Naruse, R. Studies on the Ice Sheet Flow and Local Mass Budget in Mizuho Plateau, Antarctica. *Contributions from the Institute of Low Temperature Science* **28**, 1-54 (1979).

69 Manson, R., Coleman, R., Morgan, P. & King, M. Ice velocities of the Lambert Glacier from static GPS observations. *Earth, planets and space* **52**, 1031-1036 (2000).

70 Zhang, S. K. *et al.* Ice velocity from static GPS observations along the transect from Zhongshan station to Dome A, East Antarctica. *Ann. Glaciol* **48**, 113-118 (2008).

71 Frezzotti, C., Alessandro, C. & Vittuari, L. Comparison between glacier ice velocities inferred from GPS and sequential satellite images. *Ann. Glaciol* **27**, 54-60 (1998).

72 Brecher, H. Photographic determination of surface velocities and elevations on Byrd Glacier. *Antarctic Journal of the United States* **17**, 79-81 (1982).

73 Chuter, S. J. & Bamber, J. L. Antarctic Ice Shelf Thickness from Cryosat-2 Radar Altimetry. *Geophys. Res. Lett.* **42**, 975-980 (2015).

74 Blankenship, D., Kempf, S. & Young, D. IceBridge HiCARS 1 L2 Geolocated Ice Thickness, http://nsidc.org/data/IR1HI2 (2011).

75 Blankenship, D., Kempf, S. & Young, D. IceBridge HiCARS 2 L2 Geolocated Ice Thickness, http://nsidc.org/data/IR2HI2 (2012).

76 Allen, C. IceBridge MCoRDS L3 Gridded Ice Thickness, Surface, and Bottom, http://nsidc.org/data/IRMCR3 (2013).

77 Allen, C. Pre-IceBridge MCoRDS L2 Ice Thickness, http://nsidc.org/data/BRMCR2 (2011).

78 Scambos, T. A., Dutkiewicz, M. J., Wilson, J. C. & Bindschadler, R. A. Application of Image Cross-Correlation to the Measurement of Glacier Velocity Using Satellite Image Data. *Remote sensing of environment* **42**, 177-186 (1992).

79 Bindschadler, R. A. & Scambos, T. A. Satellite-Image-Derived Velocity-Field of an Antarctic Ice Stream. *Science* **252**, 242-246 (1991).

80 Leprince, S., Barbot, S., Ayoub, F. & Avouac, J. P. Automatic and Precise Orthorectification, Coregistration, and Subpixel Correlation of Satellite Images, Application to Ground

Deformation Measurements. *IEEE Transactions on Geoscience & Remote Sensing* **10**, 1529-1558 (2007).

81 Eliason, E. M. & Mcewen, A. S. Adaptive Box Filters for Removal of Random Noise from Digital Images. *Photogramm Eng Rem S* **56**, 453-458 (1990).

82 Wessel, P., Smith, W. H. F., Scharroo, R., Luis, J. & Wobbe, F. Generic Mapping Tools: Improved Version Released. *Eos Transactions American Geophysical Union* **94**, 409-410 (2013).

83 Allison, I. & Hyland, G. Amery Ice Shelf compiled and merged ice thickness datasets, https://data.aad.gov.au/metadata/records/AIS_thickness_bottom (2010).

84 Fretwell, P. *et al.* Bedmap2: improved ice bed, surface and thickness datasets for Antarctica. *The Cryosphere* **7**, 375-393 (2013).

85 Young, D. A. *et al.* A dynamic early East Antarctic Ice Sheet suggested by ice-covered fjord landscapes. *Nature* **474**, 72-75 (2011).

86 van Wessem, J. M. *et al.* Improved representation of East Antarctic surface mass balance in a regional atmospheric climate model. *J Glaciol* **60**, 761-770 (2014).

87 Ligtenberg, S. R. M., Helsen, M. M. & van den Broeke, M. R. An improved semi-empirical model for the densification of Antarctic firn. *The Cryosphere* **5**, 809-819 (2011).

88 Zwally, H., Giovinetto, M., Beckley, M. & Saba, J. Antarctic and Greenland Drainage Systems, http://icesat4.gsfc.nasa.gov/cryo_data/ant_grn_drainage_systems.php (2012).

89 van Wessem, J. M. *et al.* The modelled surface mass balance of the Antarctic Peninsula at 5.5 km horizontal resolution. *The Cryosphere* **10**, 271-285 (2016).

90 Lenaerts, J. T. M. *et al.* Extreme Precipitation and Climate Gradients in Patagonia Revealed by High-Resolution Regional Atmospheric Climate Modeling. *J Climate* **27**, 4607-4621 (2014).

91 Lenaerts, J. T. M., Broeke, M. R. v. d., Berg, W. J. v. d., Meijgaard, E. v. & Munneke, a. P. K. A new, high-resolution surface mass balance map of Antarctica(1979 – 2010) based on regional atmospheric climate modeling. *Geophys. Res. Lett.* **39**, L04501 (2012).

92 Brecher, H. Surface velocity determination on large polar glaciers by aerial photogrammetry. *Ann. Glaciol* **8**, 22-26 (1986).

93 Pritchard, H. D. *et al.* Antarctic ice-sheet loss driven by basal melting of ice shelves. *Nature* **484**, 502-505 (2012).

94 Nakayama, Y., Schröder, M. & Hellmer, H. H. From circumpolar deep water to the glacial meltwater plume on the eastern Amundsen Shelf. *Deep Sea Research Part I: Oceanographic Research Papers* **77**, 50-62 (2013).

95 Jacobs, S. S., Jenkins, A., Giulivi, C. F. & Dutrieux, P. Stronger ocean circulation and increased melting under Pine Island Glacier ice shelf. *Nature Geosci.* **4**, 519-523 (2011).

96 Thomas, R., Rignot, E., Kanagaratnam, P., Krabill, W. & Casassa, G. Force-perturbation analysis of Pine Island Glacier, Antarctica, suggests cause for recent acceleration. *Ann. Glaciol* **39**, 133-138 (2004).

97 Rignot, E. *et al.* Recent Antarctic ice mass loss from radar interferometry and regional climate modelling. *Nature Geosci.* **1**, 106-110 (2008).

98 Rignot, E., Mouginot, J. & Scheuchl, B. MEaSUREs InSAR-based Ice Velocity of the Amundsen Sea Embayment, http://nsidc.org/data/nsidc-0545.html (2013).

99 Hillenbrand, C. D. *et al.* Grounding-line retreat of the West Antarctic Ice Sheet from inner

Pine Island Bay. *Geology* **41**, 35-38 (2013).

100 Jenkins, A. *et al.* Observations beneath Pine Island Glacier in West Antarctica and implications for its retreat. *Nature Geosci.* **3**, 468-472 (2010).

101 Vaughan, D. G. *et al.* Recent rapid regional climate warming on the Antarctic Peninsula. *Climatic change* **60**, 243-274 (2003).

102 Cook, A., Fox, A., Vaughan, D. & Ferrigno, J. Retreating glacier fronts on the Antarctic Peninsula over the past half-century. *Science* **308**, 541-544 (2005).

103 Martinson, D. G., Stammerjohn, S. E., Iannuzzi, R. A., Smith, R. C. & Vernet, M. Western Antarctic Peninsula physical oceanography and spatio–temporal variability. *Deep Sea Research Part II Topical Studies in Oceanography* **55**, 1964-1987 (2008).

104 Rignot, E. *et al.* Accelerated ice discharge from the Antarctic Peninsula following the collapse of Larsen B ice shelf. *Geophys. Res. Lett.* **31**, L18401 (2004).

105 Hulbe, C. L., Scambos, T. A., Youngberg, T. & Lamb, A. K. Patterns of glacier response to disintegration of the Larsen B ice shelf, Antarctic Peninsula. *Global Planet. Change* **63**, 1-8 (2008).

106 Scambos, T. A., Bohlander, J. A., Shuman, C. A. & Skvarca, P. Glacier acceleration and thinning after ice shelf collapse in the Larsen B embayment, Antarctica. *Geophys. Res. Lett.* **31**, L18402 (2004).

107 Wouters, B. *et al.* Dynamic thinning of glaciers on the Southern Antarctic Peninsula. *Science* **348**, 899-903 (2015).

108 Mengel, M. & Levermann, A. Ice plug prevents irreversible discharge from East Antarctica. *Nature Clim. Change* **4**, 451-455 (2014).

109 Cook, C. P. *et al.* Dynamic behaviour of the East Antarctic ice sheet during Pliocene warmth. *Nature Geosci.* **6**, 1-5 (2013).

110 Greenbaum, J. *et al.* Ocean access to a cavity beneath Totten Glacier in East Antarctica. *Nature Geosci.* **8**, 294-298 (2015).

111 Rintoul, S. R. *et al.* Ocean heat drives rapid basal melt of the Totten Ice Shelf. *Sci Adv* **2**, e1601610 (2016).

112 Li, X., Rignot, E., Mouginot, J. & Scheuchl, B. Ice flow dynamics and mass loss of Totten Glacier, East Antarctica from 1989 to 2015. *Geophys. Res. Lett.* **43**2016).

113 Aitken, A. R. A. *et al.* Repeated large-scale retreat and advance of Totten Glacier indicated by inland bed erosion. *Nature* **533**, 385-389 (2016).

114 McMillan, M. *et al.* Increased ice losses from Antarctica detected by CryoSat-2. *Geophys. Res. Lett.* **41**, 3899-3905 (2014).
